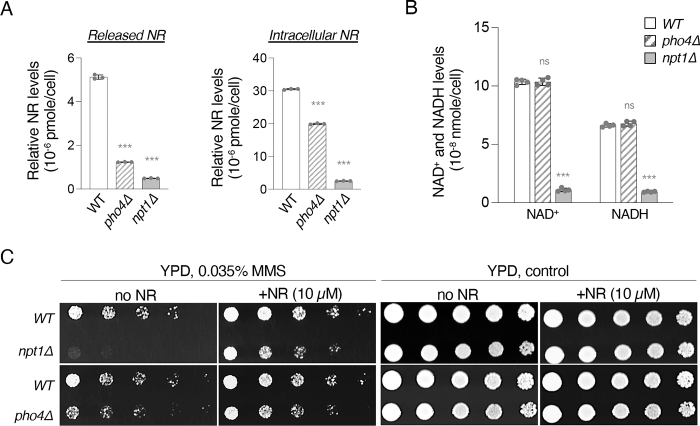# The Pof1 nicotinamide mononucleotide adenylyl transferase has a non-canonical role in NAD^+^ metabolism in the budding yeast *Saccharomyces cerevisiae*

**DOI:** 10.1016/j.jbc.2026.111456

**Published:** 2026-04-16

**Authors:** Yi-Ching Lee, Chi-Chun Huang, Matilda McDaniel, Katie Huang, Lan-Hsuan Lee, Gordon Lao, Darshan Karthi, Su-Ju Lin

**Affiliations:** Department of Microbiology and Molecular Genetics, College of Biological Sciences, University of California, Davis, California, USA

**Keywords:** cell metabolism, NAD^+^ biosynthesis, nucleotide metabolism, replication stress, yeast genetics, yeast metabolism

## Abstract

The regulation of NAD^+^ metabolism and its interaction with other cellular pathways are not yet completely understood. Here we study why cells lacking the Pof1 NMNAT have high levels of nicotinamide riboside (NR), produced from NAD^+^ turnover and in response to nutritional stress. To understand the mechanism, we identify novel NR-producing factors using genetic screens and further characterize Phm8 as an NMN nucleotidase. In addition, loss of Pof1 moderately delays the turnover of Sdt1, another nucleotidase contributing to NR production. Interestingly, the ATPase domain of Pof1 appears to play a minor role in NR production compared with the NAD^+^ synthesis catalytic domain. We further study another NMNAT, Nma1, that also contains the conserved NAD^+^ synthesis domain. Nma1 overexpression only partially reduces NR production in *pof1Δ* cells, implying the NAD^+^ synthesis domain of Pof1 may affect ATPase activity or NMN binding. Moreover, an inverse correlation between the expression of NMNATs and nucleotidases under nutritional stress also contributes to *pof1Δ*-associated NR production. Supporting this, NR production is enhanced upon moderate glucose depletion, while Pof1 protein is reduced. Overall, our data suggest that in *pof1Δ* cells, NMN is likely more accessible to the nucleotidases, resulting in NR production. Additionally, the similarity between NR production and nucleotide degradation indicates that NR production is likely important under replication stress. We show that NR levels positively correlate with resistance to replication stress. Our findings help understand the critical role of Pof1 in modulating NR production and the putative role of NR in overall nucleotide metabolism.

Nicotinamide adenine dinucleotide (NAD^+^) and its derivatives are essential coenzymes participating in a variety of redox reactions, including glycolysis, the tricarboxylic acid cycle, fatty acid oxidation, and mitochondrial oxidative phosphorylation. Besides its redox role in energy metabolism, NAD^+^ is a co-substrate for enzymes involved in epigenetic regulation (1, 2, 3), DNA repair (4), and RNA capping (5, 6). Due to its extensive impacts on cellular processes, dysregulated NAD^+^ metabolism is associated with many pathological conditions (7, 8, 9), and supplementation of NAD^+^ precursors has been shown to have ameliorating effects in many disease models (10, 11, 12, 13). To date, the mechanisms of the regulation of NAD^+^ metabolism are not completely understood due to the dynamic flexibility of NAD^+^ metabolic pathways as well as the complex interconnections with other cellular processes and signaling networks.

NAD^+^ biosynthesis in yeast is maintained through *de novo* synthesis from the amino acid tryptophan or the salvage of NAD^+^ precursors, including nicotinic acid (NA), nicotinamide (NAM), and nicotinamide riboside (NR) (Fig. 1*A*). In the *de novo* pathway, Bna proteins catalyze enzymatic reactions leading to the production of quinolinic acid (QA) (14). Bna6 then converts QA into nicotinic acid mononucleotide (NaMN), which is also made in the NA-NAM salvage branch. In the standard growth medium with ample NA, the NA-NAM salvage branch is the preferred route for NAD^+^ synthesis. In this branch, NAM is deaminated to NA by Pnc1 (15). Subsequently, Npt1 catalyzes the conversion of NA into NaMN. The enzymes nicotinamide mononucleotide adenylyl transferases (NMNATs) (16, 17), Nma1 and Nma2, convert NaMN into nicotinic acid adenine dinucleotide (NaAD), which is then amidated to NAD^+^ by the glutamine-dependent NAD^+^ synthetase Qns1 (18). The NR salvage branch may integrate with NA-NAM salvage when NR is turned into NAM by nucleosidases, Urh1 and Pnp1 (19, 20). NR can also be phosphorylated by Nrk1 (21) to yield nicotinamide mononucleotide (NMN). The NMNATs, Nma1, Nma2, and Pof1, convert NMN to NAD^+^ by transferring the adenylyl moiety of ATP to NMN (16, 17, 22).

**Figure 1:**
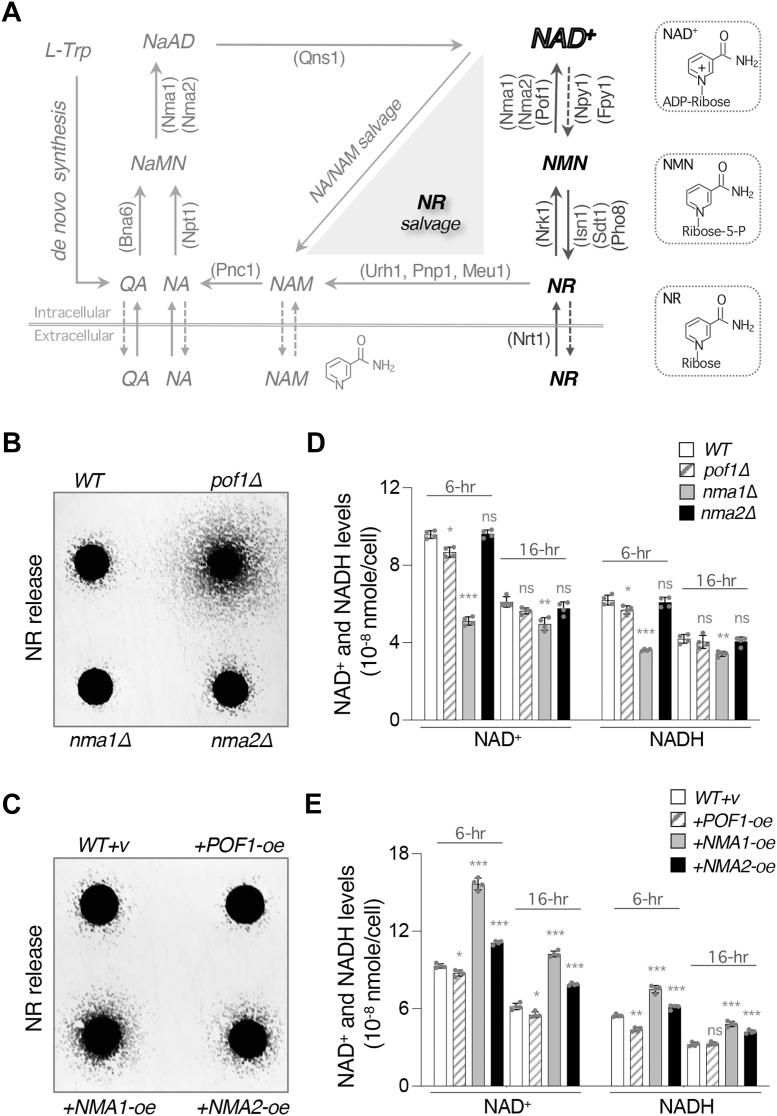
**Studying the impacts of NMNATs on NR and NAD^+^levels.***A*, a simplified model of the NAD^+^ biosynthetic pathways in *Saccharomyces cerevisiae*. In the NR salvage branch, cells can import NR through the NR transporter Nrt1. NR can be converted to NMN by Nrk1. NMN is then converted to NAD^+^ by the three NMNATs Nma1, Nma2, and Pof1. The pyrophosphatases Npy1 and Fpy1 convert NAD^+^ to NMN, and the nucleotidases Isn1 and Sdt1 as well as the phosphatase Pho8 converts NMN to NR. NR also contributes to the NA-NAM salvage branch through conversion to NAM. The *de novo* synthesis branch converges with NA-NAM salvage at the production of NaMN. Both Nma1 and Nma2 can convert NaMN into NaAD, which is made into NAD^+^ by Qns1. Abbreviations of NAD^+^ intermediates are *italicized*. *NA*, nicotinic acid; *NaAD*, deamido-NAD^+^; *NAM*, nicotinamide; *NaMN*, nicotinic acid mononucleotide; *NMN*, nicotinamide mononucleotide; *NR*, nicotinamide riboside; *QA*, quinolinic acid; *TRP*, tryptophan. Abbreviations of protein names are shown in parentheses. Nrk1, NR kinase; Isn1 and Sdt1, nucleotidase; Pho8, phosphatase; Nrt1, NR transporter; Urh1, Pnp1, and Meu1, nucleosidase; Pnc1, nicotinamidase; Npt1, nicotinic acid phosphoribosyl transferase; Bna6, quinolinic acid phosphoribosyl transferase; Qns1, glutamine-dependent NAD^+^ synthetase. *B*, comparisons of relative NR release in cells lacking *POF1*, *NMA1* or *NMA2*. Deletion of *POF1* (*pof1Δ*) significantly increases NR release whereas *nma1Δ* cells show decreased NR release. Relative NR release is determined using a NR-specific cross-feeding reporter assay in which the NR-dependent recipient cells receive NR released from the four feeder spots and grow into microcolonies around the feeder spots. *C*, cells overexpressing *POF1* (*POF1*-oe) show decreased NR release, whereas cells with *NMA1*-oe or *NMA2*-oe show increased NR release. *D*, comparisons of the NAD^+^ and NADH levels in cells lacking *POF1*, *NMA1* or *NMA2* in 6-h (nutrient-abundant) and 16-h (nutrient-deprived) cultures. A small yet significant reduction in the NAD^+^(H) levels is shown in the *pof1Δ* mutant. The *nma1Δ* mutant displays a more dramatic decrease in the NAD^+^(H) levels. *E*, *POF1*-oe cells display a slightly reduced NAD^+^ level, whereas *NMA1*-oe and *NMA2*-oe cells exhibit a higher NAD^+^ level. For *B* and *C*, feeder cell spots along with recipient cells (*bna6Δnpt1Δpho5Δ*) were grown on YPD plates at 30 °C for 4 days. The experiments were repeated four times with a total of eight biological replicates. Images shown are representative of the trend observed. For *C* and *E*, each of the NMNATs is overexpressed by the *ADH1* promoter. For *D* and *E*, the graphs are representative of the trend observed across three independent experiments (representing six biological replicates). Error bars represent data from two biological replicates, each with two technical replicates. The *p* values are calculated using Two-Way *ANOVA*. (∗, *p* < 0.05; ∗∗, *p* < 0.01; ∗∗∗, *p* < 0.005; ns, not significant).

NMNATs are considered key NAD^+^ biosynthetic enzymes because they are required in both *de novo* and salvage pathways. We previously identified Pof1 as an NMNAT in yeast (22). Unlike the other two NMNATs, Nma1 and Nma2, which show dual-substrate specificity towards both NaMN and NMN (16), Pof1 exhibits single-substrate specificity towards NMN (22). Interestingly, cells lacking *POF1* release significant levels of NR, which is a unique phenotype not seen in cells lacking *NMA1* or *NMA2* (22). Despite their central roles in NAD^+^ biosynthesis, studies have shown that NMNATs also exhibit additional functions. For example, Nma1 and Nma2 have been shown to attenuate proteotoxicity in yeast models of proteinopathies (23). Pof1 may play a role in protein quality control through its ATPase activity (24). In flies and mice, NMNAT exerts neuronal protection *via* its chaperone activity (25, 26, 27). Indeed, NMNATs are emerging therapeutic targets for a number of diseases (10, 28), highlighting the significance of elucidating their diverse functions.

Studies have shown that multiple nutrient-sensing pathways are connected to NAD^+^ homeostasis (29), which allows cells to adjust metabolic reactions and adapt to environmental changes. NAD^+^ *de novo* synthesis is associated with copper sensing (30) and external adenine sensing pathways (31). NR production is induced by nutrient depletion, and its regulation is associated with the phosphate (Pi) sensing signaling (*PHO*) pathway (32) and the extracellular amino acid-sensing SPS (Ssy1-Ptr3-Ssy5) pathway (33). Pnc1, a key enzyme in the NA-NAM salvage branch, has been shown to respond to many stress conditions, including glucose depletion and DNA damage (34, 35). However, whether NMNATs respond to specific nutrient signals remains elusive, and the regulation of the NMNATs at the transcriptional and post-transcriptional levels is still incompletely understood. In this study, we characterize the regulation of NMNATs with a focus on Pof1-associated NR metabolism. We also investigate whether altered NR metabolism may impact overall nucleotide metabolism under stress. Moreover, we identify factors that contribute to the prominent increase of NR production in cells lacking *POF1*. Our results help further understand the multifaceted roles of NMNATs in modulating NAD^+^ homeostasis.

## Results

### The levels of NR and NAD^+^ positively correlate with the dosage of NMNATs except for Pof1

To further understand the function of each of the NMNATs, we first examined the effects of their gene deletions and overexpression on NR and NAD^+^ levels. Using an NR-specific cross-feeding reporter system (22, 32, 36, 37), relative NR release levels were compared among these strains (Fig. 1, *B* and *C*). In this system, feeder strains of interest release NR to support the growth of the NR-dependent recipient cells, which manifests as microcolonies radiating from the feeder spots (22, 32, 36, 37). Consistent with previous findings, cells lacking *POF1* released a significant amount of NR (Fig. 1*B*) (22). On the contrary, the *nma1Δ* mutant showed a reduction in NR release compared to the wildtype (*WT*) control, while the *nma2Δ* mutant showed no significant difference (Fig. 1*B*). We also examined whether overexpression (*oe*) of NMNATs would influence NR release. Both *NMA1-oe* and *NMA2-oe* significantly increased NR release, with *NMA1-oe* showing a higher increase (Fig. 1*C*). Conversely, *POF1*-*oe* slightly reduced NR release (Fig. 1*C*). It is unclear why *POF1-oe* reduces NR level; however, this is in line with *pof1Δ* cells showing higher NR release (Fig. 1*B*).

Next, we asked whether the differences observed in NR release correlate with cellular NAD^+^ levels. We determined NAD^+^(H) levels in both 6-h and 16-h cultures to include the effects of NMNATs in both nutrient-abundant (6-h, actively growing early logarithmic phase) and nutrient-deprived (16-h, late logarithmic phase) conditions. Figure 1*D* showed that deletion of *NMA1* significantly decreased NAD^+^(H) levels in both growth conditions, whereas deletion of *NMA2* had no significant effect. Deletion of *POF1* only slightly decreased NAD^+^(H) levels in 6-h culture (Fig. 1*D*). As for NMNAT overexpression, *NMA1-oe* and *NMA2-oe* significantly increased NAD^+^(H) levels with *NMA1-oe* having a stronger effect (Fig. 1*E*). To our surprise, *POF1-oe* slightly decreased NAD^+^(H) levels (Fig. 1*E*). Overall, our analyses of NAD^+^(H) levels are in line with previous studies, showing that Nma1 is the major NMNAT (22, 38). Additionally, our results support a positive correlation between the dosage of *NMNATs* and cellular levels of NR and NAD^+^. This correlation, however, does not appear to be the case for *POF1*. We showed that both *pof1Δ* and *POF1-oe* slightly decreased cellular NAD^+^ level. It is plausible that *pof1Δ* has a minor impact on NAD^+^ synthesis since Pof1 is a less efficient NMNAT and that Pof1 only contributes to NAD^+^synthesis through the NR salvage pathway (22) (Fig. 1*A*). Nevertheless, the high NR release phenotype of the *pof1Δ* cells cannot be justified by its minor impact on NAD^+^ synthesis. Our results collectively indicate that Pof1 may have a noncanonical yet unique role in NR metabolism.

### Phm8, Sdt1 and Isn1 together contribute to the cytoplasmic pool of NR production in *pof1Δ* cells

**Figure 2:**
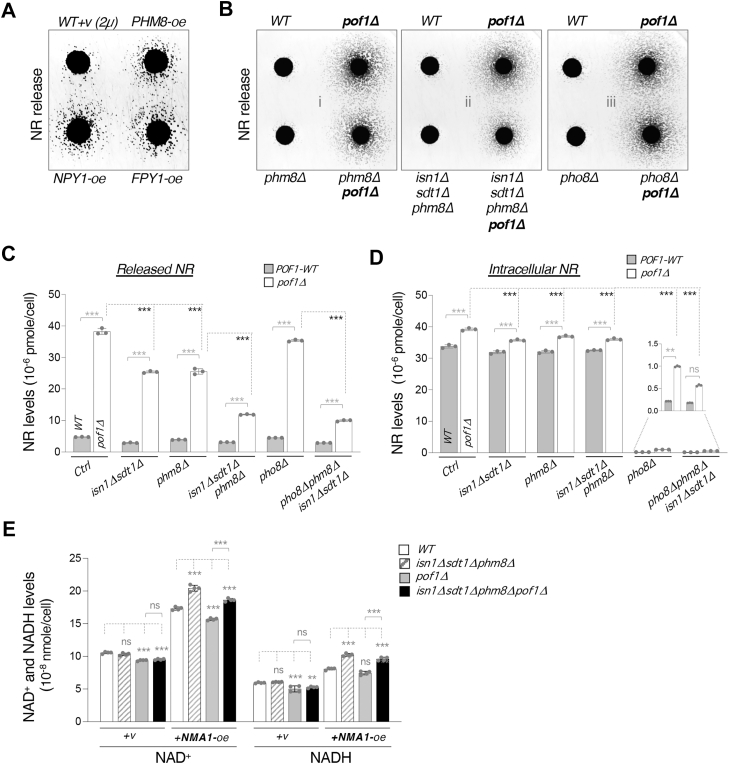
**Determination of the contribution of multiple nucleotidases to NR production in cells lacking *POF1*.***A*, cells overexpressing *PHM8*, *NPY1*, or *FPY1* show increased NR release. Gene overexpression is driven by the native promoter in the episomal *2μ*-based plasmids. *B*, deletion of *ISN1*, *SDT1*, and *PHM8* (*isn1Δsdt1Δphm8Δ*) significantly reduces NR release in *pof1Δ* cells. *C*, quantitative analysis of released NR in the growth media. Deletions of *ISN1*, *SDT1*, and *PHM8* individually or in combination significantly reduce NR release in *pof1Δ* cells with *isn1Δsdt1Δphm8Δ* displaying the strongest effect. *D*, quantitative analysis of NR in the cell lysates. Deletion of *PHO8* significantly reduces the intracellular NR levels in both WT and *pof1Δ* strains. *E*, deletion of *ISN1*, *SDT1*, and *PHM8* further enhances NAD^+^(H) levels in the *NMA1*-oe background with and without *POF1*. For *A* and *B*, feeder cell spots along with recipient cells (*bna6Δnpt1Δpho5Δ*) were grown on YPD + G418 (for *A*, G418 is added to retain the *2μ* overexpression plasmids) or YPD (for *B*) plates at 30 °C for 3 days. The experiments were repeated three times with a total of six biological replicates. Images shown are representative of the trend observed. For *C–E*, the graphs are representative of the trend observed across three independent experiments representing three (for *C* and *D*) or six (for *E*) biological replicates. For *C* and *D*, error bars represent data from three technical replicates. For *E*, error bars represent data from two biological replicates, each with two technical replicates. The *p* values are calculated using two-way *ANOVA*. (∗, *p* < 0.05; ∗∗, *p* < 0.01; ∗∗∗, *p* < 0.005; ns, not significant).

To further understand the role of Pof1 in NR metabolism, we next sought to identify factors that contribute to the increased NR production in *pof1Δ* cells. The vacuolar phosphatase Pho8 (22) and cytoplasmic pyridine 5′-nucleotidases Isn1 and Sdt1 (39, 40) have been shown to convert NMN to NR. Although deletions of *PHO8, ISN1, and SDT1* substantially reduced the intracellular NR levels in *pof1Δ* and *WT* cells, *pof1Δ* cells lacking these three enzymes still released a significant amount of NR (22). This result suggests that additional factors also contribute to NR production. To identify additional NR-producing factors, we carried out a genetic screen searching for gene overexpression that could increase NR release in *WT* cells. Among the 4524 genes examined, only four genes passed two rounds of confirmation tests: *NMA1*, *NPY1*, *FPY1*, and *PHM8* (Fig. 2*A*). (Note that *SDT1* was not present in the library.) Identification of *NMA1* in the screen further validated our independent *NMA1-oe* studies shown in Figure 1*C*, supporting a positive correlation of NAD^+^ levels and NR production. Both Npy1 and Fpy1 have been shown to display NAD^+^(H) pyrophosphatase activity (41, 42), and the identification of these two genes supports the idea that enhanced NAD^+^ degradation to NMN promotes downstream NR production (Fig. 1*A*). We first focused on Phm8 because Phm8 is a paralog of Sdt1 (43), and Phm8 is the only putative nucleotidase identified in the screen. In addition, Phm8 is involved in ribonucleotide degradation (44), and it has also been suggested to be an NMN nucleotidase (39). Moreover, *PHM8* gene expression is regulated by the phosphate (Pi) sensing *PHO* pathway (45), which is associated with NR salvage (32, 46).

To further study the role of Phm8, we first deleted *PHM8* and determined its impact on NR production in both *WT* and *pof1Δ* cells and in combination with deletions of other nucleotidases (Fig. 2*B*). Deleting *PHM8* slightly reduced NR release (Fig. 2*B*, *i*). Given that Phm8 shares the same subcellular localization as Isn1 and Sdt1, we further deleted these cytoplasmic nucleotidases and examined the impact on NR production. Deleting *ISN1*, *SDT1*, and *PHM8* all together significantly blocked NR release in the *pof1Δ* background (Fig. 2*B*, *ii*), indicating all three nucleotidases contribute to the NR increase in *pof1Δ* cells. Interestingly, deleting the vacuolar phosphatase *PHO8* did not seem to affect NR release in *pof1Δ* cells (Fig. 2*B*, *iii*). It is possible that the Pho8-produced vacuolar NR pool (22) may not be readily released extracellularly and that the plate assay is not sensitive enough to reflect the changes of Pho8-produced NR. Collectively, these results also suggest that in *pof1Δ* cells, the released NR pool is mainly produced by cytoplasmic nucleotidases (Fig. 2*B*, *ii*). To further address this, we specifically determined NR levels in the growth medium (released) and in the cell lysate (intracellular). As shown in Figure 2*C*, deleting either *PHM8* alone or deleting *ISN1* together with *SDT1* (*phm8Δ* or *isn1Δsdt1Δ*) significantly reduced the released NR level to about 65% in the *pof1Δ* background. Deleting *ISN1, SDT1, and PHM8* all together further lowered the released NR level to about 30% (Fig. 2*C*). These results indicate that the released NR level correlates more closely with the NR production activity in the cytoplasm, which is governed by Isn1, Sdt1, and Phm8. In comparison, deleting *PHO8* did not significantly affect NR release (Fig. 2, *B* and *C*). Notably, deleting *PHO8* almost eliminated intracellular NR in both *WT* and *pof1Δ* cells (Fig. 2*D*), suggesting intracellular NR level correlates more closely with Pho8-mediated NR production activity in the vacuole.

We next investigated whether cells lacking *ISN1*, *SDT1*, and *PHM8* (*isn1Δsdt1Δphm8Δ)* would affect cellular NAD^+^ level. As shown in Figure 2*E*, *isn1Δsdt1Δphm8Δ* did not significantly affect the NAD^+^ level in both WT and *pof1Δ* backgrounds. It is possible that deleting these enzymes has a more significant impact under specific conditions. Since NR production appears to positively correlate with the NAD^+^ level (Fig. 1, *C* and *E*), we surmised that these NR-producing enzymes have a more significant impact when the NAD^+^ level is high. To address this, we raised the NAD^+^ level by overexpressing *NMA1* and determined the effect of *isn1Δsdt1Δphm8Δ* on the NAD^+^ level. Interestingly, the NAD^+^ levels in *NMA1-oe* cells were further increased by *isn1Δsdt1Δphm8Δ* in both WT and *pof1Δ* backgrounds (Fig. 2*E*). These results suggest that cells may convert excess NAD^+^ to NR to maintain proper NAD^+^ levels through NR salvage factors including NAD^+^(H) pyrophosphatases Npy1 and Fpy1, and nucleotidases Phm8, Isn1, and Sdt1.

### Phm8 has NMN nucleotidase activity

**Figure 3:**
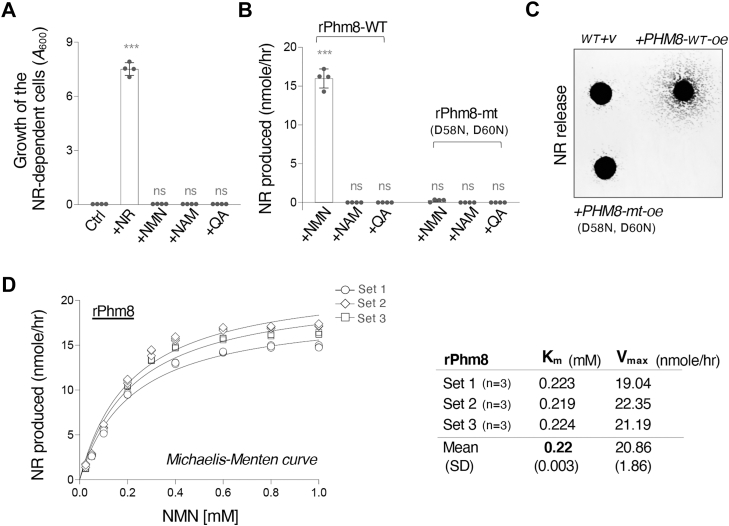
**Characterization of Phm8 as an NMN nucleotidase.***A*, NR-dependent cells (*bna6Δnpt1Δpho5Δ*) only utilize NR to support cell growth. *B*, recombinant Phm8 converts NMN to NR whereas the mutant rPhm8 cannot. *C*, cells overexpressing *PHM8* show increased NR release. Gene overexpression is driven by the *ADH1* promoter. The experiments were repeated three times with six biological replicates. Images shown are representative of the trend observed. *D*, kinetic characterization of rPhm8. The Michaelis-Menten plot shows that rPhm8 catalyzes the conversion of NMN to NR. The kinetic parameters are estimated from three biological replicates, each with three technical replicates for each condition in an experiment. For *A* and *B*, the graphs are representative of the trend observed across three independent experiments representing three biological replicates. Error bars represent data from four technical replicates. The *p* values are calculated using One-Way *ANOVA*. (∗, *p* < 0.05; ∗∗, *p* < 0.01; ∗∗∗, *p* < 0.005; ns, not significant).

Although Phm8 was suggested to be a potential NMN nucleotidase (39), this specific activity of Phm8 has not been extensively studied. To address this, we expressed recombinant Phm8 (rPhm8) in *E coli* and purified rPhm8 for enzyme kinetic analysis using NMN as a substrate. The amount of Phm8-produced NR was calculated using NR standard curves based on the growth of the NR-dependent reporter strain (*bna6Δnpt1Δpho5Δ*) (22, 32, 36). To further validate the specificity of this assay, we first confirmed that the NR-dependent cells could only utilize NR, but not other NAD^+^ precursors (NMN, NAM, and QA), for growth (Fig. 3*A*). Next, we determined whether purified rPhm8 exhibits NMN nucleotidase activity. As shown in Figure 3*B*, rPhm8 produces NR when NMN is present. We also examined whether observed NR production is associated with rPmh8 catalytic activity, and not because of NMN degradation. To address this, we constructed a catalytically deficient rPhm8 mutant and then asked whether mutating the critical catalytic residues of Phm8 would abolish its NMN nucleotidase activity. It has been reported that the two aspartate residues in the DXDXT motif are important for the phosphatase activity of Phm8 (47). We converted the two aspartate residues into asparagine to generate the catalytically deficient rPhm8 (D58N, D60N) mutant as previously described (47). As shown in Figure 3*B*, the rPhm8 (D58N, D60N) mutant failed to convert NMN to NR, suggesting that Phm8 employs the same catalytic residues to function as NMN nucleotidase. Moreover, we further confirmed that Phm8 catalytic activity is linked to NR production activity in *vivo*. The result shown in Figure 3*C* indicates that cells overexpressing the catalytically deficient Phm8 mutant no longer release more NR. In comparison, cells overexpressing WT *PHM8* show a significant increase in NR release (Fig. 3*C*). Next, we further characterized the enzyme efficiency of rPhm8 towards NMN. The enzyme kinetic analysis was carried out using 5 μg of rPhm8 and various concentrations of NMN. Apparent *K*_*m*_ and *V*_*ma*x_ for NMN were determined to be ∼ 0.22 mM and 20.86 (nmole/hr), respectively (Fig. 3*D*). Note that this *K*_*m*_ value is close to the reported physiological NMN concentration in the cell (39), suggesting that Phm8 is an effective NMN nucleotidase.

### Pof1 facilitates Sdt1 protein turnover

**Figure 4:**
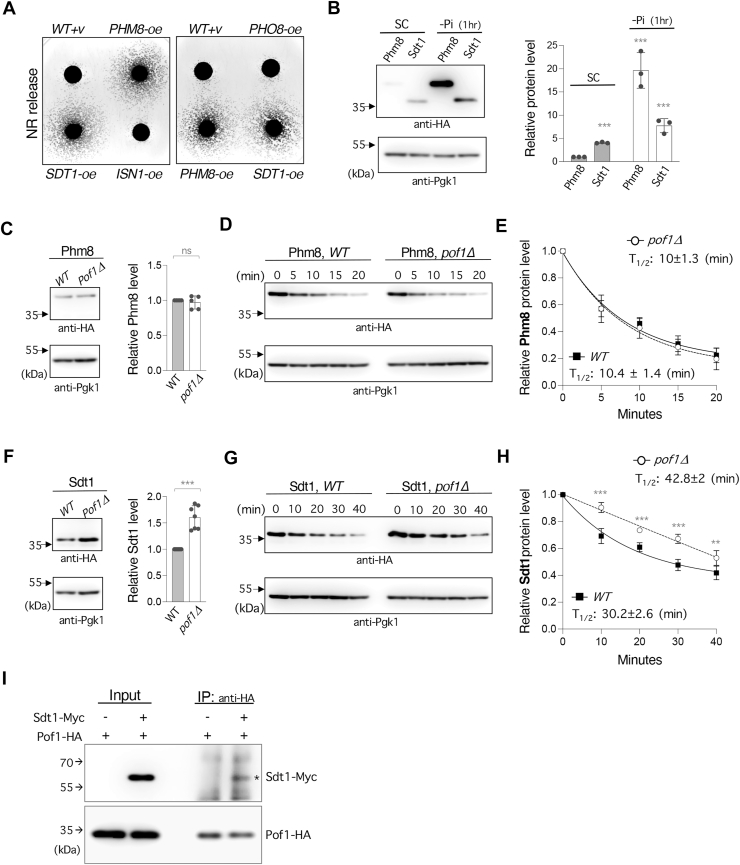
**Deletion of *POF1* moderately enhances the protein level and stability of Sdt1.***A*, cells overexpressing *PHM8* or *SDT1* show increased NR release. Gene overexpression is driven by the *ADH1* promoter. The experiments were repeated three times with six biological replicates. Images shown are representative of the trend observed. *B*, comparison of Phm8 and Sdt1 protein expression in regular and low-phosphate (Pi) conditions by Western blot analysis. HA-tagged cells were harvested either after 6-h culture in synthetic complete medium (SC) or 5-h culture in SC followed by 1-h culture in low-Pi SC. Relative protein expression is normalized to Pgk1 and the protein expression of Phm8-HA in regular SC is set to 1 (*right panel*). *C*, deletion of *POF1* does not alter the protein level of Phm8-HA. Relative protein expression is normalized to Pgk1 and the Phm8-HA level in WT cells is set to 1 (*right panel*). *D*, cycloheximide (CHX) chase studies of Phm8-HA in WT and *pof1Δ* cells. Deletion of *POF1* does not affect the rate of Phm8-HA protein turnover. *E*, quantitative analysis of the results from three independent experiments including the one shown in *D*. Relative protein expression is normalized to Pgk1 and the protein expression of Phm8-HA in WT and *pof1Δ* cells without CHX treatment is individually set to 1. *F*, deletion of *POF1* increases the protein level of Sdt1-HA. Relative protein expression is normalized to Pgk1 and the Sdt1-HA level in WT cells is set to 1 (*right panel*). *G*, deletion of *POF1* slows down Sdt1-HA protein degradation. *H*, quantitative analysis of the results from three independent experiments including the one shown in *G*. Relative protein expression is normalized to Pgk1 and the protein expression of Sdt1-HA in WT and *pof1Δ* cells without CHX treatment is individually set to 1. *I*, co-immunoprecipitation showing that Pof1 interacts with Sdt1. The image is representative of the trend observed across three independent experiments. For *B, C, and F*, quantitative analysis is shown in the *right panel* and error bar represents data from three (for *B*), five (for *C*), or seven (for *F*) biological replicates. For *E* and *H*, protein half-life is estimated from three independent experiments. Error bars represent data from three biological replicates. The *p* values are calculated using Two-Way *ANOVA* for *B* and Student’s *t* test for *C, E, F and H*. (∗, *p* < 0.05; ∗∗, *p* < 0.01; ∗∗∗, *p* < 0.005; ns, not significant).

Next, we asked whether specific NMN nucleotidases are upregulated in *pof1Δ* cells. In addition to being a NAD^+^ metabolic enzyme (22), Pof1 has also been suggested to play a role in protein quality control, and the ATPase activity of Pof1 may help unfold specific proteins to facilitate their degradation by proteasomes (24). Moreover, Pof1 has been shown to physically interact with Ubc7 (24), a ubiquitin-conjugating enzyme. It is therefore possible that Pof1 facilitates the degradation of specific NMN nucleotidases to modulate NR production, and we hypothesized that cells lacking Pof1 might have increased levels of NMN nucleotidases. To identify potential targets, we first examined how each of the NMN nucleotidases affects NR release upon overexpression. Because the *SDT1-oe* plasmid was not present in the Yeast ORF 2.0 library that we used for the genetic screen, we cloned each of the NMN nucleotidases and overexpressed them using the *ADH1* promoter. As expected, *PHM8*-oe significantly increased NR release (Fig. 4*A*) and exhibited similar NR release phenotype like the *pof1*Δ cells (Fig. 1*B*). Interestingly, *SDT1*-oe also increased NR release whereas *ISN1*-oe and *PHO8*-oe exerted little effect on NR release (Fig. 4*A*). We therefore hypothesized that deletion of *POF1* might increase the protein levels of Phm8 and/or Sdt1, which promotes the activity of NR production. Because phosphate (Pi) depletion is known to induce Phm8 expression (44, 45), we first compared the protein levels of Phm8 and Sdt1 in regular and low-Pi conditions. In regular conditions, Sdt1 appeared more abundant than Phm8 (Fig. 4*B*). Phm8 protein was substantially increased by Pi-depletion whereas Sdt1 was slightly increased (Fig. 4*B*). These results are in line with reported gene expression studies and the increased protein levels are likely due to Pho4-induced gene expression in response to Pi-depletion (44, 45). Next, we examined whether Pof1 affects the protein levels of Phm8 and Sdt1. Deletion of *POF1* had no impact on Phm8 protein level (Fig. 4*C*). Interestingly, a ∼1.5-fold increase in Sdt1 protein level was observed in *pof1Δ* cells (Fig. 4*F*). We further examined whether Pof1 affects the turnover of Phm8 and Sdt1. Cycloheximide chase was employed to stop protein translation and the levels of Phm8 and Sdt1 were monitored using Western blot analysis. Because Phm8 levels were very low under regular conditions (Fig. 4*B*), we raised Phm8 expression by Pi-depletion and examined whether deleting *POF1* would prolong its half-life. Interestingly, Phm8 had a relatively short half-life (T_1/2_: ∼10 min), which is in line with its low expression level in regular condition (Fig. 4*B*). However, deletion of *POF1* did not influence Phm8 protein half-life (Fig. 4, *D* and *E*). In comparison, Sdt1 protein half-life appeared to slightly increase in *pof1Δ* cells (42.8 ± 2 min) compared to WT cells (30.2 ± 2.6 min) (Fig. 4, *G* and *H*). Our results indicate that Pof1 facilitates the turnover of Sdt1. Admittedly, cycloheximide chase has its limitations because cycloheximide also inhibits the translation of protein degradation systems like proteasomes. To further support our model, we examined whether Pof1 interacts with Sdt1 using co-immunoprecipitation. As shown in Figure 4*I*, a small yet perceptible physical interaction between Pof1 and Sdt1 was observed. Together, our results suggest that the NR increase in *pof1Δ* cells is in part due to a decrease in Sdt1 turnover.

### The activity of NAD^+^ degradation is associated with NR production in *pof1Δ* cells

**Figure 5:**
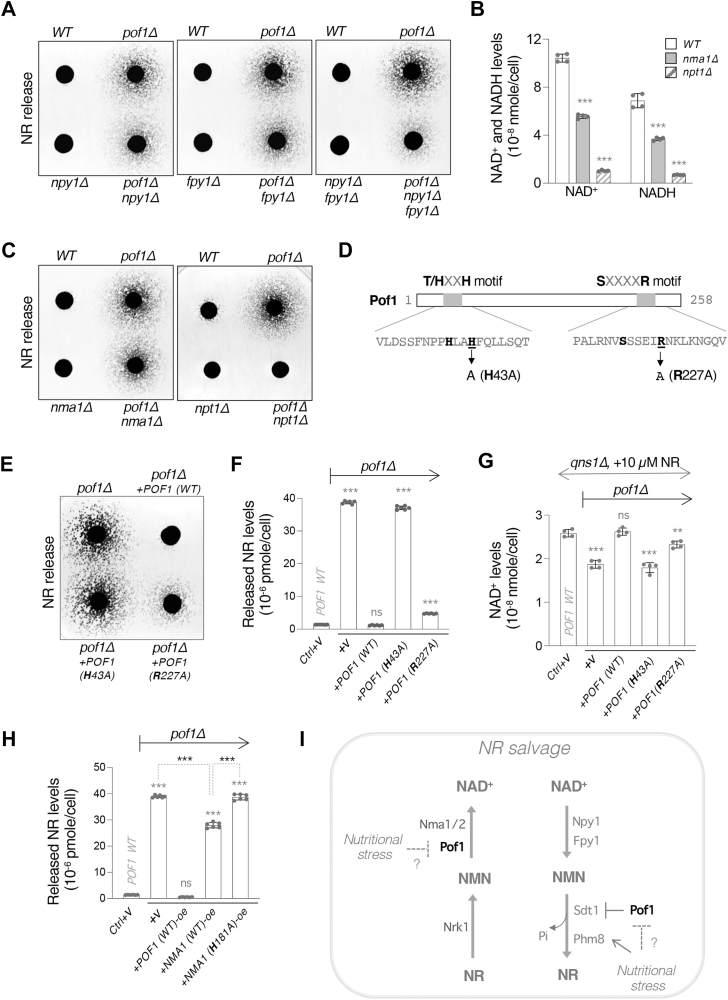
**NR production correlates with NAD^+^turnover, and Pof1 modulates NAD^+^turnover to NR.***A*, deletion of *NPY1* and *FPY1* individually or in combination significantly reduces NR release in *pof1Δ* cells with *npy1Δfpy1Δ* displaying the strongest effect. *B*, NAD^+^(H) levels decrease in both *nma1Δ* and *npt1Δ* cells, with *npt1Δ* cells showing a more dramatic decrease. *C*, deletion of *NMA1* slightly reduces NR release whereas deletion of *NPT1* eliminates NR release in *pof1Δ* cells. *D*, illustration showing the two ATP-binding motifs, T/HXXH and SXXXXR, in the Pof1 protein. *E*, the *POF1 (H43A)* mutant gene fails to reduce NR release in the *pof1Δ* cells. *F*, quantitative analysis of released NR in the growth media. Results show that the T/HXXH of Pof1 is essential in NR production. *G*, the *POF1 (H43A)* mutant gene fails to restore the NAD^+^ level in *qns1Δpof1Δ* cells. *H*, *NMA1-oe* significantly reduces NR release in *pof1Δ* cells. Gene overexpression is driven by the *ADH1* promoter. *I*, the proposed model depicting that Pof1 is critical in modulating NMN accessibility. Pof1 plays a role in converting NMN to NAD^+^. Pof1 also facilitates Sdt1 protein turnover, which reduces NMN conversion to NR. Likely, the NMNATs and the NMN nucleotidases compete for NMN accessibility. Loss of *POF1* increases the NMN accessibility to NMN nucleotidases, which further promotes NR production. For *A*, *C*, and *E*, the experiments were repeated three times with six biological replicates. Images shown are representative of the trend observed. For *B*, *F*, *G*, and *H*, graphs are representative of the trend observed across three independent experiments representing three (for *F* and *H*) or six (for B and *G*) biological replicates. Error bars represent data from two biological replicates, each with two (for *B* and *G*) or three (for *F* and *H*) technical replicates. The *p* values are calculated using One-Way *ANOVA* for *F, G, and H,* and Two-Way *ANOVA* for *B*. (∗, *p* < 0.05; ∗∗, *p* < 0.01; ∗∗∗, *p* < 0.005; ns, not significant).

Next, we further investigated the connection between NAD^+^ metabolism and NR production. In our screen for gene overexpression that increases NR release, we identified two NAD^+^(H) pyrophosphatases, *NPY1* and *FPY1* (Fig. 2*A*). These results suggested that NR production is associated with the activity of NAD^+^ degradation into NMN. We first asked whether NAD^+^ degradation plays a role in *pof1Δ*-associated NR production. As shown in Figure 5*A*, deleting *NPY1* or *FPY1* alone slightly decreased NR release whereas deleting both *NPY1* and *FPY1* together significantly reduced NR release in the *pof1Δ* background. These results implied that *pof1Δ*-associated NR production originates from NAD^+^ and the produced NMN is likely converted to NR by nucleotidases. Next, we examined whether lowering the NAD^+^ level would block *pof1Δ*-associated NR production. Nma1 and Npt1 are two major NAD^+^ biosynthetic enzymes that have been shown to have a significant impact on NAD^+^ levels (36). Figure 5*B* showed that deleting *NMA1* reduced the NAD^+^ level to about 50% in the *WT* cells, whereas deleting *NPT1* lowered the NAD^+^ level more substantially to about 10%. Interestingly, deleting *NMA1* slightly decreased NR release yet deleting *NPT1* appeared to eliminate NR release in the *pof1Δ* background (Fig. 5*C*). Together, these results indicate that *pof1Δ*-associated NR production is related to both NAD^+^ level and the activity of NAD^+^ degradation into NMN. However, unlike the case of Sdt1, the level of Npy1 and Fpy1 did not appear to be altered in *pof1Δ* cells (data not shown). Moreover, the positive correlation between NR and NAD^+^ levels was not the case for Pof1 since *POF1* deletion and overexpression only slightly affected the NAD^+^ levels (Fig. 1, *D* and *E*).

### The ATP-binding residues within the catalytic domain of Pof1 play a major role in NR production

We therefore further examined whether the catalytic activity of Pof1 plays a role in NR production. Two conserved functional domains characterized as the ATP-binding motifs, T/HXXH and SXXXXR (Fig. 5*D*), are present in NMNATs across different species (48, 49). To understand whether these two domains play a role in *pof1Δ*-associated NR production, we separately mutated the histidine residue in the T/HXXH motif (H43A) and the arginine residue in the SXXXXR motif (R227A) (Fig. 5*D*). These residues have been similarly mutated and studied for the functions of NMNATs in other species. For example, the T/HXXH motif was shown to be involved in the conversion of NMN to NAD^+^ (50, 51, 52). On the other hand, some studies suggested that the SXXXXR motif is dispensable for NAD^+^ synthesis but is related to the chaperone activity of NMNAT (53, 54). In the *pof1Δ* background, introducing a single copy of the WT version of *POF1* significantly reduced NR release (Fig. 5*E*) to the level of WT cells (Fig. 5*F*). In comparison, the *POF1 (H43A)* gene failed to reduce NR release in the *pof1Δ* background (Fig. 5, *E* and *F*). Interestingly, the *POF1 (R227A)* gene significantly reduced NR release (Fig. 5, *E* and *F*); however, it did not reduce NR to the WT level. These results suggest that both motifs are involved in *pof1Δ*-associated NR production and notably, the T/HXXH motif that governs NAD^+^ synthesis activity appears to be more important. Next, we tested whether the *POF1(H43A)* mutant had completely lost NAD^+^ synthesis activity. We employed the *qns1Δ* mutant to test the effects of these *POF1* variants on the NAD^+^ level because the impact of *POF1* on the NAD^+^ level is very subtle in the WT background (Fig. 1, *D* and *E*). Moreover, it has been shown that *POF1* deletion has a more significant impact on the NAD^+^level in the *qns1Δ* background, when NR is the sole source for NAD^+^ synthesis (22). As shown in Figure 5*G*, the *POF1* (*H43A*) mutant is indeed catalytically deficient because it failed to complement the loss of *POF1* and restore the NAD^+^ level in *qns1Δpof1Δ* cells. In comparison, the *POF1* (R227A) mutant still possesses NAD^+^ synthesis activity close to the WT level. Together, our results indicate that the T/HXXH motif of Pof1 plays a major role in preventing excess NR production and is essential for NAD^+^ synthesis. Nevertheless, the chaperone-related SXXXXR motif has a smaller yet still significant impact on NR production (Fig. 5, *E* and *F*), and it may play a role in modulating Sdt1 turnover.

It remains unclear why having the other two NMNATs (Nma1 and Nma2), which also contain the T/HXXH motif, is not sufficient to compensate for the loss of *POF1* regarding NR production. It is possible that in *pof1Δ* cells, NMN becomes more accessible to nucleotidases, which results in significant NR production. Although NMN is also accessible to the other NMNATs, competition is expected between NMNATs and nucleotidases. To further understand the mechanism, we examined whether NR production in *pof1Δ* cells could be reduced by overexpressing *NMA1*. Interestingly, *NMA1*-oe moderately reduced NR release in *pof1Δ* cells (Fig. 5*H*), and the reduction is blocked by mutating the T/HXXH motif of *NMA1* (*H181A*). In comparison, *POF1*-oe decreased NR release to the WT level (Fig. 5*H*). These results indicate that *NMA1*-oe can partially slow down NR production in *pof1Δ* cells, likely by facilitating the conversion of NMN to NAD^+^. Overall, our results suggest that Pof1 plays a role in modulating NMN accessibility and slows down NAD^+^ turnover through NR salvage (Fig. 5*I*). As a less efficient NMNAT (22), we speculate that Pof1 binding to NMN would slow down both NAD^+^ synthesis and NR production from NMN. Deletion of *POF1* hinders the conversion of NMN to NAD^+^ (Fig. 5, *G* and *I, left*), and more NMN is converted to NR by nucleotidases (Fig. 5*I, right*). Excess NR production due to the loss of *POF1* can be partially rescued by overexpressing *NMA1* (Fig. 5*H*). On the other hand, deletion of *POF1* also enhances Sdt1 protein stability, which promotes NR production. Moreover, nutritional stress could increase Phm8 expression (44, 45) to facilitate NMN conversion to NR, which is further enhanced in cells lacking Pof1.

### Pof1 protein level is downregulated upon glucose depletion

**Figure 6:**
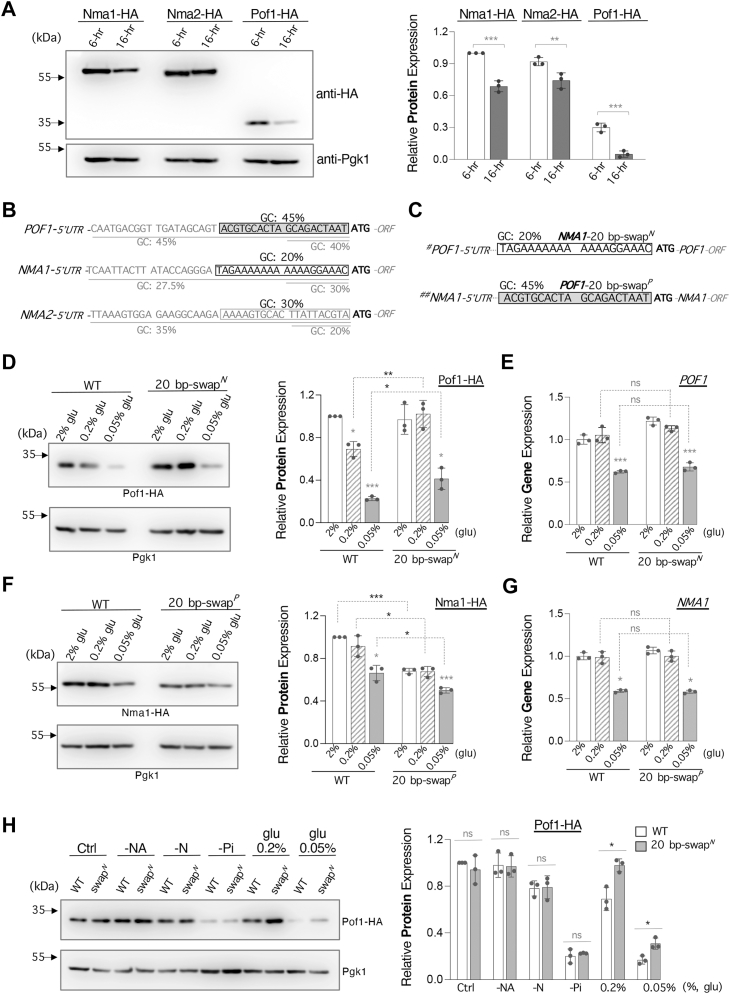
**A higher GC content in the 5′ untranslated region of *POF1* contributes to the reduction of Pof1 protein in low-glucose conditions.***A*, comparison of NMNAT protein expression in two growth stages. HA-tagged cells were cultured in YPD for 6 or 16 h. Pof1-HA shows a significant reduction in the 16-h culture. Relative protein expression is normalized to Pgk1 and the Nma1-HA level at 6 h is set to 1 (*right panel*). *B*, comparison of the 5′ untranslated region (5′ UTR) of each *NMNAT*. *POF1* possesses a relatively higher GC content (45%) in the first 20 nucleotides of the 5′ UTR compared with the 5′ UTR of *NMA1* (20%) and *NMA2* (30%). *C*, illustration showing the 5′ UTR sequences of *POF1* and *NMA1* after swapping their first 20 nucleotides immediately before the start codon. *Top: POF1* ORF with *NMA1 5′ UTR “20 bp-swap*^*N*^*”. Bottom: NMA1* ORF with *POF1 5′ UTR “20 bp-swap*^*P*^*”. D*, expression analysis of Pof1-HA in WT and the 20 bp-swapped strains. Pof1 protein shows a decrease in 0.2% and 0.05% glucose in WT strain. Swapping the 20 nucleotides increases Pof1-HA protein level in low-glucose conditions. Pof1-HA protein level is normalized to Pgk1 and the Pof1-HA level of WT in SC (2% glu) is set to 1 (*right panel*). *E*, gene expression analysis of *POF1* in WT and 20 bp-swapped strains determined by qPCR. Swapping the 20 nucleotides does not alter *POF1* mRNA expression patterns in different glucose conditions. *F*, swapping the 20 nucleotides reduces Nma1-HA protein level. Nma1-HA protein level is normalized to Pgk1 and the Nma1-HA level of WT in SC (2% glu) is set to 1 (*right panel*). *G*, swapping the 20 nucleotides does not alter *NMA1* mRNA expression patterns. *H*, swapping the 20 nucleotides only increases Pof1-HA protein expression in low-glucose conditions. Pof1-HA protein level is normalized to Pgk1 and the Pof1-HA level of WT in SC (Ctrl) is set to 1 (*right panel*). Abbreviations: -NA, low-nicotinic acid; -N, low-nitrogen source (ammonium sulfate); -Pi, low-phosphate; glu, glucose. For *A*, *D*, *F*, and *H*, quantitative analysis is shown in the *right panel* and error bars represent data from three biological replicates. For *E* and *G*, the graphs are representative of the trend observed across three independent experiments representing three biological replicates. Relative gene expression is normalized to *TAF10* and the gene expression of WT in SC is set to 1. Error bars represent data from three technical replicates. The *p* values are calculated using Two-Way *ANOVA* except for *H*, which is determined by Student’s *t* test. (∗, *p* < 0.05; ∗∗, *p* < 0.01; ∗∗∗, *p* < 0.005; ns, not significant).

**Figure S1:**
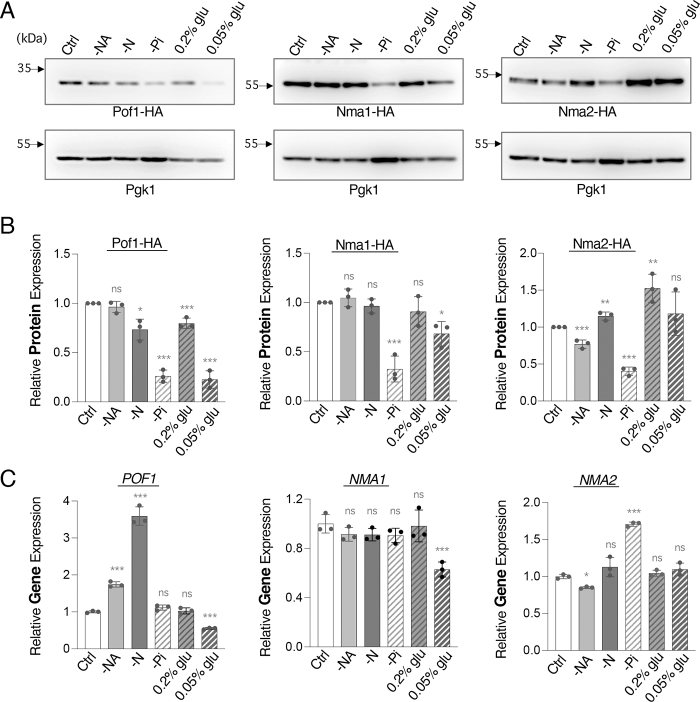


NR production is positively correlated with the activation of phosphate (Pi)-sensing *PHO* pathway (32). Given that both Phm8 and Sdt1 are major NR-producing factors (Fig. 4*A*) and that their protein levels are increased in response to Pi-depletion (Fig. 4*B*), we asked whether Pof1 expression is also regulated by Pi-depletion and other nutrient deprivations. First, we compared the expression of Pof1 along with Nma1 and Nma2 under nutrient-abundant (6-h) and nutrient-deprived (16-h) conditions by Western blot analysis of HA-tagged NMNATs. The levels of Pof1 protein were overall lower than Nma1 and Nma2 in both 6-h and 16-h cultures (Fig. 6*A*), and significant decreases were observed for each of the NMNATs in the 16-h culture compared with the 6-h culture. Interestingly, the level of Pof1 showed a more significant decrease (reduced to ∼13%, with Pof1 level at 6-h set as 100%) while Nma1 and Nma2 showed moderate decreases to ∼69% and ∼80%, respectively (Fig. 6*A*). This suggests Pof1 may be more sensitive to specific nutrient deprivations. To address this, we determined the protein levels of each of the NMNATs in specific nutrient-deprived conditions (Fig. S1). Our results showed that the protein levels of all NMNATs were significantly reduced in the low-Pi condition (Fig. S1, *A* and *B*). These results support that reduced NMNAT expression facilitates NMN conversion to NR (Fig. 5*I*) upon Pi-depletion, which is in line with the study showing that the activation of the Pi-sensing pathway promotes NR production (32). Interestingly, compared with Nma1 and Nma2, Pof1 appeared to be more sensitive to glucose depletion. In low-glucose conditions (0.2% and 0.05% glucose), a more significant drop was observed in Pof1 protein level (Fig. S1, *A* and *B*). We also examined that the observed Pof1 protein reduction was not simply due to decreased gene expression (Fig. S1*C*). Overall, the results indicate that apart from gene expression regulation, additional factors also contribute to the sharp reduction of Pof1 protein in low-glucose conditions. Further studying how glucose depletion regulates Pof1 protein expression may help understand the differences between Pof1 and the other two NMNATs.

Glucose starvation has been shown to destabilize the translation initiation complex and inhibit the protein synthesis of a wide range of genes (55). Interestingly, the GC nucleotide content of the 5′ UTR immediately upstream of the start codon was suggested to inversely correlate with translation efficiency upon glucose starvation (56). Genes that are translationally unaffected by glucose starvation likely have 34 to 35% GC in the first 70 nucleotides of 5′ UTR while genes that are translationally upregulated have lower GC content (56). Upon examining the 5′ UTR of NMNATs, we found that the 5′ UTR of *POF1* possesses a relatively higher overall GC content (45%), while the GC% of the 5′ UTR of *NMA1* and *NMA2* are 20% and 30%, respectively (Fig. 6*B*). To examine whether the high GC content of the 5′ UTR of *POF1* plays a role in the reduction of Pof1 protein level in low-glucose conditions, we replaced the 20 nucleotides immediately upstream of the start codon of *POF1* with the corresponding region of *NMA1* (Fig. 6*C*), and *vice versa* using CRISPR. We chose *NMA1* for two reasons. For the 20 nucleotides immediately upstream of the start codon, *NMA1* possesses the lowest GC content (20%) (Fig. 6*B*). Additionally, *NMA1* shared a similar alteration in gene expression with *POF1* in low-glucose conditions (Fig. 6, *E* and *G*). In line with our expectations, replacing the first 20 nucleotides of the 5′ UTR of *POF1* with that of *NMA1* significantly increased Pof1 protein level in 0.2% glucose (Fig. 6*D*), with no significant impact on the transcript level (Fig. 6*E*). Because *POF1* gene expression was downregulated in 0.05% glucose (Fig. 6*E*), swapping the 20 nucleotides only exerted a smaller but significant increase in the protein level of Pof1 (Fig. 6*D*). In comparison, replacing the first 20 nucleotides of the 5′ UTR of *NMA1* with the corresponding region of *POF1* significantly decreased Nma1 protein level in all glucose conditions (Fig. 6*F*). *NMA1* gene expression pattern was not altered by the 5′ UTR swap (Fig. 6*G*). Overall, these results support that the higher GC content in the 5′ UTR of *POF1* reduces the translation efficiency during glucose deprivation. In addition, swapping the 20 nucleotides of 5′ UTR only enhanced the protein level of Pof1 in low-glucose conditions but not in other nutrient-deprived conditions (Fig. 6*H*), suggesting that the effect of 5′ UTR is specific to glucose deprivation-induced translational regulation.

### NR production is increased in response to moderate glucose restriction and correlates with MMS resistance

**Figure 7:**
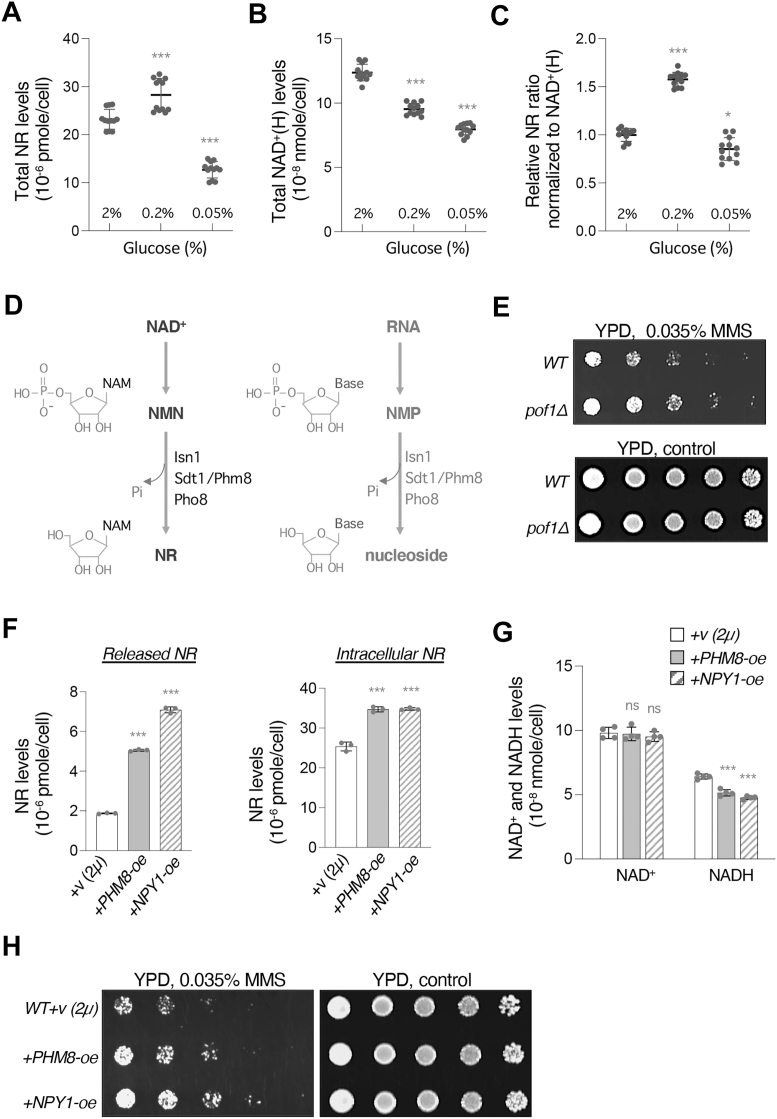
**Cellular NR level is increased during moderate glucose depletion, and it also affects MMS resistance.***A*, total NR levels are increased in cells grown in 0.2% glucose but decreased in cells grown in 0.05% glucose. *B*, total NAD^+^(H) levels are decreased in cells grown in low-glucose conditions. *C*, the relative NR ratio is elevated in cells grown in 0.2% glucose. The relative NR ratio is the total NR levels normalized to total NAD^+^(H) levels and the relative NR ratio in regular condition (2% glucose) is set to 1. *D*, an illustration showing the similarities between NAD^+^ and RNA degradation. The nucleotidases Isn1, Sdt1, Phm8, and Pho8 convert nicotinamide mononucleotide (NMN) to NR. Isn1, Sdt1, Phm8 and Pho8 can also degrade nucleoside monophosphate (NMP) into nucleoside. *E*, comparison of MMS sensitivity. Result shows that *pof1*Δ cells are more resistant to MMS. *F*, *PHM8*-oe and *NPY1*-oe strains display an increase in released NR and intracellular NR levels. *G*, *PHM8*-oe and *NPY1*-oe strains display a regular NAD^+^ level but a reduced NADH level. *H*, *PHM8*-oe and *NPY1*-oe strains exhibit greater resistance to MMS. For *E* and *H*, serial dilutions (five-fold) of the indicated strains were spotted onto YPD (for *E*) or YPD + G418 (for *H*) plates with 0.035% MMS. Cells were grown on plates at 30 °C for 2 days. The experiments were repeated three times with a total of six biological replicates. Images shown are representative of the trend observed. For *A*, *B*, and *C*, graphs are based on data from four independent experiments. Error bars represent data from four biological replicates, each with three technical replicates. For *F*, *G* and *H*, *PHM8* and *NPY1* are overexpressed using the *2μ*-based plasmids and the expression is driven by the native promoter. G418 is added to the growth medium to retain the *2μ*-based plasmids. For *F* and *G*, graphs are representative of the trend observed across three independent experiments representing three (for *F*) or six (for *G*) biological replicates. For *F*, error bars represent data from three technical replicates. For *G*, error bars represent data from two biological replicates, each with two technical replicates. The *p* values are calculated using One-Way *ANOVA* for *A, B, C and F,* and Two-Way *ANOVA* for *G*. (∗, *p* < 0.05; ∗∗, *p* < 0.01; ∗∗∗, *p* < 0.005; ns, not significant).

The connection of Pof1 protein reduction with glucose deprivation (Fig. 6*D*) as well as the unique NR release phenotype of the *pof1Δ* mutant (Fig. 1*B*) prompted us to further investigate whether NR production contributes to other cellular processes that are sensitive to glucose deprivation. To address this, we first examined whether NR production is increased in response to glucose deprivation by determining the total NR levels (the sum of intracellular NR and released NR) in regular and low-glucose conditions. Interestingly, total NR levels were elevated only in cells grown in 0.2% glucose but not in cells grown in 0.05% glucose (Fig. 7*A*). Because NR levels correlate with NAD^+^(H) levels (Fig. 1, *C* and *E*), cells grown in 0.05% glucose may have lower NAD^+^(H) levels. We therefore also determined the total NAD^+^(H) levels in these cells. Figure 7*B* showed that the total NAD^+^(H) levels declined as the glucose concentration decreased. To factor in the differences of NAD^+^(H) levels, we normalized the total NR levels to the total NAD^+^(H) levels and obtained relative NR ratios with the ratio of cells grown in 2% glucose set to 1. As shown in Figure 7*C*, the relative NR ratio of cells grown in 0.2% glucose is still significantly increased (∼1.5 fold) compared to 2% glucose. On the other hand, the relative NR ratio of cells grown in 0.05% glucose is still less than in 2% glucose, but the difference becomes much smaller (Fig. 7*C*). This result implies that NR production is enhanced under moderate glucose depletion and that extreme glucose starvation is likely to limit NAD^+^(H) synthesis to an extent that restricts NR production.

Interestingly, Phm8, Isn1, Sdt1, and Pho8 have also been shown to be involved in the turnover of ribonucleotides (44, 57, 58). The similarity between NAD^+^ and RNA degradation (Fig. 7*D*) suggests that the conversion of NMN to NR may play a role in supporting ribonucleotide salvage. In addition, cells grown in low-glucose conditions appear to be more resistant to methyl methanesulfonate (MMS) (59), a DNA alkylating agent that causes DNA damage. Since NR production was increased in response to moderate glucose restriction (Fig. 7, *A* and *C*), we speculated that NR metabolism might be important for cell survival under replication stress and that *pof1Δ*-associated NR production might play a role. Supporting this, *pof1Δ* cells were more resistant to MMS compared to the WT cells (Fig. 7*E*). We next directly addressed whether increasing the activity of NR production is sufficient to enhance MMS resistance. To test this, we attempted to increase NR production by overexpressing *PHM8* or *NPY1*. Both *PHM8-oe* and *NPY1-oe* significantly increased the levels of released NR and intracellular NR (Fig. 7*F*). Moreover, *PHM8-oe* and *NPY1-oe* did not significantly alter the NAD^+^ level and only caused a small decrease in NADH level (Fig. 7*G*), likely because cells still have functional NMNATs. In line with our expectations, both *PHM8-oe* and *NPY1-oe* promoted MMS resistance (Fig. 7*H*), with *NPY1-oe* having a stronger effect than *PHM8-oe*. Since *NPY1-oe* produces more NR than *PHM8-oe* (Fig. 7*F*), these results further support that increased NR production alleviates sensitivity towards MMS. To further strengthen the association between NR metabolism and MMS resistance, we tested whether cells with defective NR production would show sensitivity to MMS. Both *pho4Δ* and *npt1Δ* cells displayed lower levels of released NR and intracellular NR (Fig. S2*A*). Deletion of *PHO4* did not alter the NAD^+^ level, whereas deletion of *NPT1* substantially reduced NAD^+^ level (Fig. S2*B*). In line with our expectation, both *npt1Δ* and *pho4Δ* cells were sensitive to MMS, and NR supplementation enhanced MMS resistance in *npt1Δ, pho4Δ* and *WT* cells (Fig. S2*C*). Overall, a positive correlation between NR levels and MMS resistance was observed in the studies. Our results also suggest a putative role of NR in nucleotide metabolism.

## Discussion

In this study, we investigate the role of Pof1 in NR production. We also identify and study the roles of several factors involved in NR production, including the NMNAT Nma1, the NAD^+^(H) pyrophosphatases Npy1 and Fpy1, as well as the NMN nucleotidases Phm8 and Sdt1. Our studies provide an overall positive correlation between NR production and NAD^+^ synthesis activity. Supporting evidence includes NR and NAD^+^ levels being both increased in *NMA1*-oe cells (Fig. 1, *C* and *E*) and reduced in *nma1Δ* (Fig. 1, *B* and *D*) cells. Moreover, NR production in *pof1Δ* cells is eliminated by deleting the major NAD^+^ synthesis enzyme *NPT1* (Fig. 5*C*). We also show that Npy1, Fpy1 (Fig. 5*A*), Phm8, Sdt1, and Isn1 (Fig. 2, *B* and *C*) mediate NR production in *pof1Δ* cells. Together, these results suggest that NR production is associated with NAD^+^ turnover and that Pof1 plays a role in modulating this process.

The high NR release phenotype of *pof1Δ* cells (Fig. 1*B*) suggests a crucial role for Pof1 in NR production and we show that the T/HXXH motif of Pof1 plays a major role (Fig. 5, *E* and *F*). The T/HXXH motif has been characterized as the catalytic motif for NMNATs to synthesize NAD^+^ (50, 51, 52). Compared with the other yeast NMNATs (16, 17, 60), recombinant Pof1 protein displays low apparent catalytic activity (22), implying that Pof1 is not an efficient enzyme that converts NMN to NAD^+^. Nevertheless, Pof1 is as important as other NMNATs when cells solely rely on the NR-NMN salvage pathway for NAD^+^ synthesis. Supporting this, we previously showed that deleting *POF1* lowers the NAD^+^ level to the same extent as deleting *NMA1* in the *qns1Δ* background (22). Interestingly, even though both Pof1 and Nma1 contain the T/HXXH motif that converts NMN to NAD^+^, overexpressing *NMA1* only partially reduces the NR production in *pof1Δ* cells whereas overexpressing the mutant *NMA1* (H181A) has no effect (Fig. 5*H*). These results suggest that Pof1 is a unique NMNAT that also modulates NR production. Likely, loss of Pof1 not only hinders NMN conversion to NAD^+^ but also promotes NMN degradation to NR. Supporting this, loss of Pof1 lowers the NAD^+^ levels in *NMA1*-oe cells (Fig. 2*E*). Moreover, we show that Pof1 may modulate NMN conversion to NR through its impact on Sdt1 turnover (Fig. 4, *G* and *H*). Together, our data suggest that Pof1 modulates NAD^+^ metabolism by slowing down NAD^+^ turnover to NR.

On the other hand, the SXXXXR motif of Pof1 has a less significant impact on NR production (Fig. 5, *E* and *F*). The SXXXXR motif has been linked to the chaperone activity of NMNATs (53, 54). Our data demonstrate that compared with the T/HXXH motif, the SXXXXR motif is less important in NAD^+^ production (Fig. 5*G*). It is likely that the SXXXXR motif determines the ATPase activity of Pof1, which affects Sdt1 turnover. Although other studies have suggested the two ATP-binding motifs are separately involved in either NAD^+^ synthesis activity or chaperone activity, we cannot rule out the possibility that the T/HXXH domain may also assist ATP binding for the ATPase activity of Pof1. Overall, our data suggest that both ATP-binding motifs contribute to the maintenance of proper NR production, and it appears that Pof1 is critical in modulating NMN accessibility. However, NMN binding residues are currently not well understood, and a potential NMN binding site remains elusive in yeast NMNATs (49). The ATP-binding motifs bind to the phosphate groups of ATPs (61). Given that both ATP and NMN contain phosphate groups and share structural similarities, the ATP-binding motifs may also assist NMN binding. Further studies elucidating the NMN binding site of yeast NMNATs will help clarify the unique role of Pof1 in overall NAD^+^ metabolism.

**Figure 8:**
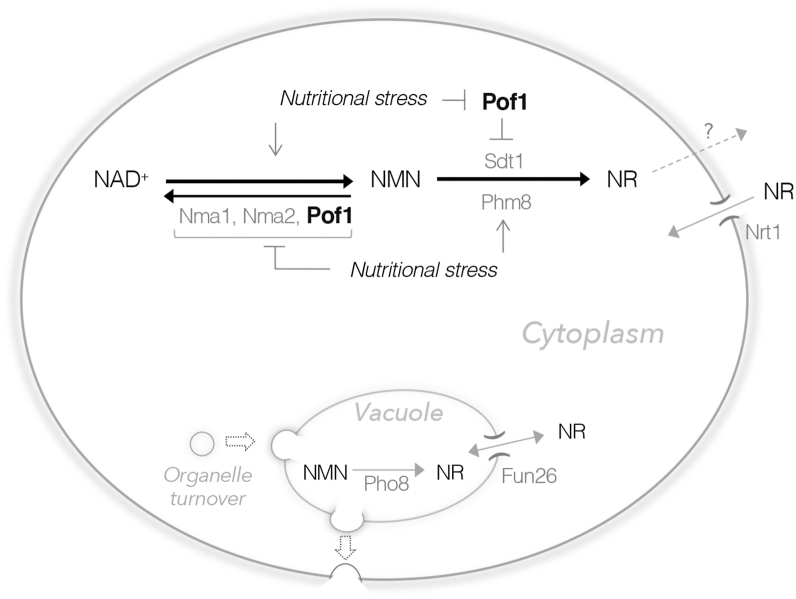
**A proposed model depicting NR homeostasis and the roles of Pof1 in NR metabolism.** NR is produced in two major compartments: cytoplasm and vacuole. Pho8 catalyzes NR production in the vacuole whereas Sdt1 and Phm8 catalyze NR production in the cytoplasm. The equilibrative nucleoside transporter, Fun26, helps balance NR between the two pools. Yeast cells release NR possibly through vesicle trafficking, and the released NR can re-enter the cell *via* the NR transporter, Nrt1. Pof1 plays a role in modulating NMN accessibility and slows down NAD^+^ turnover to NR. As a less efficient NMNAT, Pof1 binding to NMN may slow down both NAD^+^ synthesis and NR production from NMN. Nutritional stress oppositely regulates NMNATs and Phm8. Pof1 also facilitates the turnover of Sdt1. In the absence of *POF1* or under nutritional stress, NMN is likely more accessible to nucleotidases, resulting in elevated NR production.

Our studies also show that NR production is modulated by NMN nucleotidases localized in different intracellular compartments (Fig. 8). Pho8 resides inside the vacuole and is the major factor contributing to the intracellular NR pool (Fig. 2*D*), whereas the activity of the cytoplasmic enzymes, Isn1, Sdt1, and Phm8, closely correlates with the level of released NR (Fig. 2*C*). These results also suggest that cells may modulate the cytoplasmic and the vacuolar NR pools through cytoplasmic and vacuolar nucleotidases, respectively. Although we showed that in *pof1Δ* cells, the high NR release mainly originates from the cytoplasmic pool (Fig. 2*B*, *ii*), the vacuolar NR pool may also play a role since the equilibrative nucleoside transporter Fun26 (32) can function to balance the two NR pools. It remains unclear how cells release NR, but cells can uptake extracellular NR through the NR transporter Nrt1 (62) (Fig. 8). Moreover, we showed that overexpression of *PHM8* or *SDT1* is sufficient to increase the released NR level (Fig. 4*A*), suggesting Phm8 and Sdt1 are both limiting factors of NR production. Interestingly, both Phm8 and Sdt1 contribute to the high NR production in *pof1Δ* cells (Fig. 2*C*); however, we only observed a higher protein level of Sdt1 in *pof1Δ* cells (Fig. 4*F*). Our data suggest that Pof1 modulates NR production in part through its impact on Sdt1 protein turnover (Fig. 4, *G* and *H*). Meanwhile, Phm8 also contributes to the high NR production in *pof1Δ* cells (Fig. 2, *B* and *C*). Together, these findings suggest that Pof1 may modulate NMN accessibility and that NR production can be increased by activating two critical NMN nucleotidases. First, cells can modulate NR production in a Sdt1-dependent manner. Sdt1 is more abundant than Phm8 in regular growth conditions (Fig. 4*B*) and has a relatively longer half-life (Fig. 4, *E* and *H*). Therefore, Sdt1 may play a more important role in NR production to assist NAD^+^ turnover in regular growth conditions. Supporting this, deleting *SDT1* alone is sufficient to lower the intracellular NR level while deleting *PHM8* has little impact on WT cells (39). Because *SDT1* gene expression barely alters in various stress conditions (44), it is likely that Sdt1 is mostly regulated post-transcriptionally. In this study, we showed that deletion of *POF1* increases the protein level of Sdt1 (Fig. 4*F*) and prolongs its half-life (Fig. 4, *G* and *H*). On the other hand, Phm8 is another critical NMN nucleotidase whose expression is regulated by transcription factors that respond to nutrient depletion, such as Pho4 (63) and Gcn4 (64). At the transcript level, *PHM8* is highly induced by Pi-depletion as well as other stress conditions including glucose depletion, whereas *SDT1* is only slightly induced (44, 45). Our protein study of Phm8 and Sdt1 also supports that Phm8 is an inducible nucleotidase, activated in response to Pi-depletion (Fig. 4*B*). Thus, cells can also modulate NR production in a Phm8-dependent manner, which is likely activated by multiple nutrient-sensing signaling pathways. Supporting this, NR production is positively correlated with the activation of Pi-sensing *PHO* pathway (32). Moreover, moderate glucose depletion increases NR production as well (Fig. 7*A*). Collectively, our results suggest that the cytoplasmic NR production is modulated by Sdt1 as well as Phm8, and their expression can be regulated by Pof1 and nutritional stress, respectively (Fig. 8).

Because deletion of *POF1* hinders the conversion of NMN to NAD^+^ (Fig. 5*G*), NMN likely becomes more accessible to both Sdt1 and Phm8 in the absence of Pof1. Although NMN is also accessible to the other two NMNATs, competitions are expected between NMNATs and nucleotidases. We showed that overexpressing *NMA1* further increases NAD^+^ level in the absence of nucleotidases (Fig. 2*E*). Additionally, NMNATs require ATP to synthesize NAD^+^ from NMN. Therefore, NMN is more likely to be converted to NR by nucleotidases in nutrient-deprived conditions. Supporting this, we show that cells oppositely regulate NMNATs and nucleotidases in response to nutrient depletion (Fig. 8). The protein levels of NMNATs are mostly downregulated in nutrient-depleted conditions (Figs. 6*A* and S1). Notably, all NMNATs are downregulated in low-Pi conditions, and Pof1 is specifically downregulated in low-glucose conditions (Fig. S1, *A* and *B*). Conversely, Phm8 is induced by nutrient depletion (Fig. 4*B*) (44, 45). These results suggest that under nutritional stress, NAD^+^ synthesis is likely impeded and NMN is more likely to be converted to NR. It has been shown that NR production is induced by nutrition depletion (32, 33). Moreover, NAD^+^ is suggested to serve as a metabolic “sink” that helps maintain Pi homeostasis (65). In line with this, the conversion of NMN to NR by nucleotidases releases the Pi group from NMN (Figs. 1*A* and 5*I*). The inverse correlation of NMNATs and Phm8 expressions in response to nutrient depletion, such as Pi and glucose depletion, may help cells adapt to stress by coordinating NAD^+^ metabolism and NR production. Additionally, recent studies have shown that nutritional stress may induce the formation of stress granules or biomolecular condensates that could affect signaling and adaptation (66, 67, 68). It would be interesting to determine whether loss of Pof1 facilitates the formation of specific biomolecular condensates under nutritional stress and whether the nucleotidases have enhanced activity or preferential access to NMN in this environment, thereby enhancing NR production.

Because Pof1 is specifically downregulated by glucose depletion (Fig. 6), we further examined the connection of NR metabolism with other cellular processes that are sensitive to glucose depletion. We found that the NR level seems to correlate with MMS resistance. Cells with higher NR levels, including the *pof1Δ*, *NPY1*-oe, and *PHM8*-oe strains, appear to be more resistant to MMS (Fig. 7, *E* and *H*). In contrast, *npt1Δ* and *pho4Δ* cells exhibiting low NR levels are hypersensitive towards MMS (Fig. S2*C*). The sharing of enzymes between NAD^+^ and RNA degradation (Fig. 7*D*) allows coordination between these two processes. For example, *PHM8-oe* may confer MMS resistance by accelerating both NAD^+^ and RNA degradation. Moreover, NR production is likely to increase upon replication stress as the protein levels of Phm8 and Npy1 are upregulated in MMS treatments (69). NR in the cytoplasm is expected to be more dynamic and flexible. If the cytoplasmic NR is not transported into the vacuole for storage (32) or assimilated into NAD^+^, it could be excreted or shared with other cellular pathways such as ribonucleotide salvage. Stored NR in the vacuole can enter the cytoplasm and *vice versa via* an equilibrative nucleoside transporter Fun26 (32) (Fig. 8). Moreover, we showed that NR supplementation was able to enhance MMS resistance in low-NR mutant cells (*e.g.*, *pho4Δ* and *npt1Δ*) as well as in WT cells (Fig. S2*C*).

On the other hand, NAM, another NAD^+^ precursor, is also reported to confer MMS resistance (35). Unlike NR supplementation (Fig. S2*C*), NAM supplementation does not effectively restore MMS resistance in *npt1Δ* cells (35), suggesting NR and NAM may affect MMS resistance by different mechanisms. Moreover, NAM-induced MMS resistance is achieved with 5 mM NAM (35), whereas 10 μM NR was used in our study. The different concentrations used in NR and NAM supplementations suggest that NR-induced MMS resistance is unlikely to be solely mediated by NAM. We however cannot rule out this possibility since NR supplement can also be converted to NAM by nucleosidases Urh1 and Pnp1 (19). Compared with NAM, NR contains a ribose (Fig. 1*A*). It is possible that the ribose moiety in NR could be shared with ribonucleotide salvage, which helps promote MMS resistance. Nevertheless, downstream enzymes involved in the process remain to be determined. MMS is an alkylating agent that generates DNA damage, which likely results in dNTP depletion. The acceleration of NAD^+^ degradation and NR production may provide additional resources, such as ribose and Pi group, for nucleic acid synthesis to repair the damaged DNA and therefore, confer MMS resistance. Overall, this work contributes to the understanding of Pof1-mediated NR metabolism and provides insights into the association between NAD^+^ degradation, NR production, and nucleotide metabolism. Our results also support a non-canonical role for Pof1 in modulating the turnover of NAD^+^ to NR.

## Experimental procedures

### Yeast strains, growth media, and plasmids

Yeast strain BY4742 *MATα his3Δ1 leu2Δ0 lys2Δ0 ura3Δ0* acquired from Open Biosystems (70) was used as the WT strain for this study. Standard growth media including yeast extract-peptone-dextrose (YPD) and synthetic complete (SC) media were made as described (71). NA-free SC was made using niacin-free yeast nitrogen base acquired from Sunrise Science Products. Low phosphate (low-Pi) medium was prepared by phosphate precipitation from SC as previously described (32). For each liter of low-Pi SC, 2.46 g of MgSO_4_ was first dissolved in SC. Then 8 ml of concentrated ammonia was slowly added with gentle stirring to precipitate inorganic phosphate as MgNH_4_PO_4_. After filtration, HCl was added to adjust pH to 6, followed by autoclave sterilization. Gene deletions were performed using the standard PCR-based strategy to amplify gene-specific PCR products generated using either the pAG32-*hphMX4* (72) or the reusable *loxP-kanMX-loxP* (pUG6) (73) cassettes as templates. Multiple gene deletions were made by employing a galactose-inducible Cre recombinase to remove the *loxP-kanMX-loxP* cassette (73), followed by another round of gene deletion. The HA or Myc epitope tag was added to the C-terminal of target genes in the genome using the pFA6a-*3HA-HIS3MX* or pFA6a-1*3MYC-KANMX* plasmid as the template for PCR-mediated tagging (74). The NMNAT and NMN nucleotidase overexpression constructs were made in the integrative pPP81 (*LEU2*) vector as described (75), and gene overexpression is driven by the *ADH1* promoter. In brief, DNA fragments of the coding region were PCR amplified with NotI site added to the 5′ end and NheI (for *NMA1, NMA2, POF1*, and *SDT1*) or AvrII (for *PHM8*, *ISN1*, and *PHO8*) site added to the 3′ end. The DNA fragments were then cloned into pPP81 digested with NotI and NheI/AvrII. After PacI digestion, the linearized plasmids were integrated into the yeast genome as described (75). The catalytically deficient *NMA1* (H181A) overexpression plasmid was generated using the Q5 site-directed mutagenesis kit (New England Biolabs) with the primers 5′- caatcacctacttggctctaagaatgtttg -3′ and 5′- gtgaaaaagacccacatgctactattac -3’. *NPY1-oe, FPY1-oe,* and *PHM8-oe, 2 μ*-based plasmids carrying *LEU2* and *KanMX* markers, were obtained from the Dharmacon Molecular Barcoded Yeast ORF 2.0 library (Horizon, Cat#YSC11751). These episomal multi-copy *2 μ*-based plasmids were transformed into the WT strain and gene overexpression is driven by the native promoter. Cells were cultured in YPD containing 0.3 µg/ml G418 (geneticin disulfate) (Teknova, Cat# 5005) to retain the episomal *2 μ*-based plasmids. To construct WT and mutant variants of *POF1*, WT *POF1* ORF and promoter region were PCR amplified with SacI site added to the 5′ end and XhoI site added to the 3′ end. The resultant DNA fragment was then cloned into pRS306 digested with SacI and XhoI. Mutation variants of *POF1* were then generated using the Q5 site-directed mutagenesis kit (New England Biolabs). Oligos used for mutagenesis are listed as follows: *POF1* (H43A)-F, ccacatctggccgcttttcaactactat; *POF1* (H43A)-R, tggattgaatgaagaatcaaggacaaataacttctg; *POF1* (R227A)-F, gtttcatcatccgagattgcgaacaaactgaagaatgggc; *POF1* (R227A)-R, gcccattcttcagtttgttcgca atctcggatgatgaaac. After HindIII digestion, the linearized plasmids were integrated into the yeast genome at *POF1* promoter region. The bRA90 plasmid (Addgene Plasmid #100951) was used for cloning the gRNA duplex for CRISPR-mediated 5′ UTR swap studies. Cas9 is constitutively active under the *PGK1* promoter, and the gRNA duplex was cloned into the BplI site. The sequences of the gRNA duplexes for swapping the first 20 bp of the 5′ UTR of *POF1* and *NMA1* are listed as follows: *POF1*-F, aatggacacttaatcaatgagtttt; *POF1*-R, tcattgattaagtgtccattgatca; *NMA1*-F, tgcgtcaattacttataccagtttt; *NMA1*-R, tggtataagtaattgacgcagatca. Oligo duplexing was carried out in a PCR machine (100 °C for 5 min, ramps to 25 °C at 0.1 °C /sec). Duplexed oligos were then cloned into the bRA90 plasmid. All plasmid constructs were verified by DNA sequencing. The genomic DNA of CRISPR-modified strains was verified by PCR amplification and DNA sequencing.

### NR cross-feeding plate assay and measurements of NR in the growth media and cell lysate

This assay has been used as a readout for relative NR levels in yeast cells (22, 32, 36, 37, 38, 76). In brief, a specific NAD^+^ auxotrophic mutant (*bna6Δnpt1Δpho5Δ*), whose growth depends on NR, was employed as the “NR-dependent recipient cells”. This strain lacks functional *de novo* and NA-NAM salvage activities due to the deletions of *BNA6* and *NPT1*, respectively. Additionally, deleting *PHO5*, a periplasmic phosphatase, abolishes the ability of the cell to utilize NMN because NMN needs to be converted to NR before entering the cell (36). As a result, this strain can only grow when NR is supplemented. Yeast strains of interest were employed as the “NR feeder cells”. The NR-dependent recipient cells were first plated on a solid YPD agar plate (∼3 × 10^3^cells/cm^2^). Then ∼2 × 10^4^cells of each feeder cell strain were spotted onto the lawn of NR-dependent recipient cells. Plates were then incubated at 30 °C for 3 to 4 days before being photographed. Because NR is heat-labile, standard growth media do not contain NR. Therefore, the extent of the recipient cell growth indicates the levels of NR released by the feeder cells.

NR levels were determined by a liquid-based cross-feeding bioassay as previously described (22, 32, 36, 38, 46, 76) with modifications. To determine the intracellular NR levels, cells were grown in YPD for 12 to 14 h at a starting OD_600_= 0.1 to a density of OD_600_= 10. 150 OD_600_ unit cells were collected by centrifugation and lysed by bead-beating (Biospec Products) in 400 μl ice-cold 50 mM ammonium acetate solution. The supernatant was collected by centrifugation, and the pellet was extracted two more times with 600 μl ice-cold 50 mM ammonium acetate solution, which ultimately generated 1600 μl cell lysate. After filter sterilization, 40 μl (300 μl for *pho8*Δ and *npt1*Δ strains) of cell extract was used to supplement 8 ml cultures of NR-dependent recipient cells with a starting absorbance of 0.05 at 600 nm in SC. To determine the released NR levels, 20 ml supernatant of feeder cell culture was collected, filter-sterilized, and then 2 ml (4 ml for *pho4Δ* and *npt1Δ* strains) was added to NR-dependent recipient cell culture in 2× SC to a final volume of 8 ml with starting absorbance of 0.05 at 600 nm. A control culture of NR-dependent recipient cells in SC without supplementation was included in all experiments. After incubation at 30 °C for 24 h, the growth of the recipient cells (absorbance at 600 nm) was measured and normalized to the cell number of each feeder cell strain. Absorbance at 600 nm readings were then converted to concentrations of NR using the standard curves established as previously described (36).

### Measurements of cellular NAD^+^ and NADH

Total cellular levels of NAD^+^ and NADH were determined using enzymatic cycling reactions as described (75). Cells were grown in YPD for 6 h at a starting OD_600_= 0.1 to a density of OD_600_= 1.0, and 1 OD_600_ unit cells were collected in duplicate by centrifugation. Acid extraction was performed in one tube to obtain NAD^+^, and alkali extraction was performed in the other tube to obtain NADH at 60 °C for 40 min. Amplification of NAD^+^ or NADH in the form of malate was carried out using 3 μl (for NAD^+^) or 5 μl (for NADH) of neutralized acid or alkali-extracted lysate in 100 μl of cycling reaction at room temperature for 1 h. The reaction was terminated by heating at 100 °C for 5 min. Next, malate produced from the cycling reaction was converted to oxaloacetate and then to aspartate and α-ketoglutarate by the addition of 1 ml malate indicator reagent at room temperature for 20 min. The reaction produced a corresponding amount of NADH as readout, which was measured fluorometrically with excitation at 365 nm and emission monitored at 460 nm. Standard curves for determining NAD^+^ and NADH concentrations were obtained as follows: NAD^+^ and NADH were added into the acid and alkali buffer to a final concentration of 0, 2.5, and 7.5 μM, which were then treated with the same procedure along with other samples. The fluorometer was calibrated each time before use with 0, 5, 10, 20, 30, and 40 μM NADH to ensure that the detection was within a linear range.

### Screen for gene overexpression that increases NR release

The Dharmacon Molecular Barcoded Yeast ORF 2.0 library established in the BY4741 strain (77) was acquired from Horizon. In this library, a total of 4524 ORFs (including the native promoter and terminator of each gene) were cloned into a *2 μ* plasmid carrying both the *LEU2* and *KanMX* markers. To screen for strains carrying gene overexpression plasmids that increase NR release, 2 μl of each strain was directly taken from the frozen stock and then spotted onto YPD + G418 plates spread with the NR-dependent recipient cells (*npt1Δbna6Δpho5Δ*) at a density of ∼3 × 10^3^cells/cm^2^. G418 (0.3 µg/ml) is added to the medium to retain the multi-copy *2μ*-based plasmids. After incubation at 30 °C for 2 to 3 days, we scored the cross-feeding activity (which indicates the level of NR release) of each strain by comparing the diameter of the cross-feeding zone with the WT strain carrying a control *2 μ* plasmid. In the first round of genetic screens, we got 29 candidates that showed strong NR release phenotypes. These candidates were re-examined and the plasmids were extracted, amplified in *E coli*, and then subjected to DNA sequencing to confirm the identity. We re-transformed these plasmids into a different strain BY4742, re-examined the phenotypes, and found that only nine gene overexpression conferred increased NR release. Among the nine genes, *NMA1*, *NPY1*, *PHM8*, and *FPY1* showed consistent and stronger NR release. The other 5 genes, including *NMA2, VPH1, SOK1, PPH22*, and *PMA1*, either carried non-silent mutations in their ORF or showed less significant increased NR release. *SDT1* is absent in this library and therefore we did not identify *SDT1* in the screen.

### Cloning, expression, purification, and kinetic analysis of Recombinant Phm8 (rPhm8)

The *PHM8* ORF was PCR amplified from yeast genomic DNA using primers 5′-gacaggagctctatgactatcgctaaagattacagaac-3′ and 5′-aggcctcgagtgatgactgaacattgatctgttg-3’. The amplified fragment was cloned into the plasmid pET28b (Novagen) digested with SacI and XhoI. The catalytically deficient mutant *PHM8* (D58N, D60N) was generated using the Q5 site-directed mutagenesis kit (New England Biolabs) with the primers 5′- gtatttttctttaacatcaacaacactttgtacag-3′ and 5′- ttttcgagcaggatccgg-3’. The resultant plasmids pET28a-*PHM8-WT* and pET28a-*PHM8-mt* were transformed into *Escherichia coli* Rosetta (DE3) cells (Novagen), and the bacterial cells were grown in M9 medium supplemented with 50 μg/ml kanamycin and 25 μg/ml chloramphenicol. Protein induction and purification were carried out as previously described (22) with modifications. Expression of rPhm8 was induced with 0.2 mM isopropyl β-D-thiogalactopyranoside at 25 °C for 16 h. Cells were harvested by centrifugation, resuspended in 10 volumes of lysis buffer (50 mM Tris-HCl, pH 8.0, 500 mM NaCl, 5 mM imidazole, 10% glycerol, 1 mM PMSF, 1 μg/ml leupeptin, 1 mg/ml lysozyme, 1 mg/ml DNase I, 0.1% Triton X-100), and lysed by incubating in the liquid nitrogen followed by 37 °C water bath for 20 min. This freeze-thaw cycle was repeated 6 times to ensure proper cell lysis. N-terminal His_6_ tagged-rPhm8 protein was then purified using the nickel-nitrilotriacetic acid agarose resin (Genesee, Cat # 20–512). The column was washed with buffer (50 mM Tris-HCl, pH 8.0, 500 mM NaCl) containing 30 mM imidazole. Recombinant Phm8 was eluted with buffer containing 250 mM imidazole. Recombinant Phm8 elution was collected and separated into eight tubes, each with 750 μl eluate. Before storage at −20 °C, 250 μl of 100% glycerol was added to each fraction. Protein concentrations in each fraction were determined using the Bradford assay (Bio-Rad, Hercules), comparing against a BSA standard curve. The fractions were also examined by SDS-PAGE followed by Coomassie staining. A prominent rPhm8 band (∼40 kD) appeared in the eluted fractions, and the fraction that showed the least background bands (fraction 5 or 6) was used for the activity assay.

Recombinant Phm8 activity was determined using the conditions described for alkaline phosphatase activity (NEB, M0525S) with modifications. Briefly, 5 μg of rPhm8 was added to 100 μl of the reaction mix (50 mM potassium acetate, 20 mM Tris-acetate, 10 mM magnesium acetate, 100 μg/ml BSA, pH 7.9) containing various concentrations of NMN and incubated for 1 h at 30 °C. NR produced in the reaction was subsequently determined by a liquid-based cross-feeding bioassay as described above (22, 46, 76). In brief, all the reaction mix was used to supplement 8 ml cultures of NR-dependent recipient cells (*npt1Δbna6Δpho5Δ*) with a starting absorbance of 0.05 at 600 nm in SC. After incubation at 30 °C for 24 h, the growth of the recipient cells (absorbance at 600 nm) was measured and the absorbance readings were then converted to NR concentrations as previously described (22, 46, 76) using the NR standard curves (36). The kinetic parameters for NMN were estimated from the results of three independent measurements using GraphPad Prism 10.

### Immunoblot analysis

Cells were grown in YPD, SC, or nutrient-deprived media for 5 to 6 h at a starting OD_600_= 0.1 to a density of OD_600_= 1.0, and 15 OD_600_ unit cells were collected by centrifugation. Collected cells were snap-frozen in liquid nitrogen or immediately subjected to extraction. Protein extracts were obtained by bead-beating (Biospec Products) in lysis buffer: 50 mM Tris–HCl, pH 7.5, 100 mM NaCl, 1% Triton X-100, 5 mM EDTA (pH = 8), 1 mM PMSF, and protease inhibitor cocktail (Pierce). Protein concentrations were estimated using the Bradford assay (Bio-Rad, Hercules, CA, USA), comparing against a BSA standard curve, and 10 μg (Pgk1) or 15 μg (NMNATs/Sdt1/Phm8) of total protein was loaded in each lane. After electrophoresis, the protein was transferred to a polyvinylidene fluoride membrane (GE Healthcare, Amersham, UK). Blocking was carried out using OneBlock Western-CL Blocking buffer (Genesee, Cat# 20–313) or EveryBlot Blocking Buffer (Bio-Rad, Cat# 12010020) at room temperature for 1 h. The membranes were then washed and blotted with either rabbit anti-HA antibody (Cell Signaling, Cat# 3724S, 1:2000 dilution), mouse anti-PGK antibody (Invitrogen, Cat# 459250, 1:5000 dilution), or mouse anti-Myc antibody (Millipore, Cat# 05–724, 1:2000 dilution) for overnight incubation at 4 °C. Protein was visualized using anti-mouse or anti-rabbit immunoglobulin antibody conjugated to horseradish peroxidase (Invitrogen, Cat#31430 and #31460, 1:10,000 dilution) and the ECL reagents (Amersham, GE). For each protein, multiple images were taken with multiple exposure times (10 s increments) to avoid signal saturation. The chemiluminescent image was analyzed using the Amersham Imager 600 (GE) system and software provided by the manufacturer.

### Cycloheximide chase analysis

For cycloheximide chase studies, cells were grown in 40 ml SC for 5 h at a starting OD_600_= 0.1 to a density of OD_600_= 0.8 before either harvesting (0-h time point) or treating with 0.4 mg/ml cycloheximide (CHX) followed by harvesting at indicated time points as previously described (38). Some modifications were made for Phm8 studies to increase expression: cells were first grown in SC for 2 to 4 h then switched to low-Pi SC for 1 to 4 h to induce expression of Phm8. Approximately 5 OD_600_ unit cells were harvested by centrifugation, snap-frozen in liquid nitrogen or immediately subjected to extraction and Western blot analysis as described above. Protein half-lives were estimated from the results of three independent measurements using GraphPad Prism 10.

### Co-immunoprecipitation (Co-IP) analysis

Cells were grown in SC for 6 h at a starting OD_600_= 0.1 to a density of OD_600_= 1.0, and 50 OD_600_ unit cells were collected by centrifugation. Collected cells were snap-frozen in liquid nitrogen or immediately subjected to extraction. Protein extracts were obtained by bead-beating (Biospec Products) in lysis buffer: 50 mM Na–HEPES, pH 7.5, 200 mM NaOAc, pH 7.5, 1 mM EDTA (pH = 8), 1 mM EGTA, 5% glycerol, 0.25% NP-40, 3 mM DTT, 1 mM PMSF, and protease inhibitor cocktail (Pierce). The whole cell lysates (input) were drawn off the beads and centrifuged at a maximum speed (13,200 rpm) for 20 min at 4 °C. The whole cell lysates were then mixed with 50 μl anti-HA magnetic beads (Thermo Fisher Scientific) and incubated overnight at 4 °C with gentle agitation. The beads were washed three times with 1 ml 0.05% TBST to remove unbound proteins. The IP samples were eluted from the beads using 100 μl SDS sample buffer and incubated at 100 °C for 10 min. The collected IP samples and input samples were then subjected to Western Blot analysis.

### Quantitative PCR (qPCR) analysis of gene expression levels

Cells were grown in SC or nutrient-deprived media for 6 h at a starting OD_600_= 0.1 to a density of OD_600_= 1.0, and 40 OD_600_ unit cells were collected by centrifugation. Total RNA was extracted using GeneJET RNA purification Kit (Thermo Fisher Scientific), and complementary DNA was synthesized using QuantiTect Reverse Transcription kit (Qiagen) according to the manufacturer’s instructions. Approximately 50 ng of cDNA and 500 nM of specific primer sets were used for each qPCR reaction. qPCR was run in 96-well plates on Roche LightCycler 480 using LightCycler 480 SYBR green I Master Mix (Roche) as previously described (22). The average size of the amplicon for each gene was approximately 150 bp. The target mRNA transcript levels were normalized to the internal control *TAF10* transcript levels.

### MMS spot assay

Yeast strains of interest (from freshly grown YPD plates) were diluted in sterile water to an OD_600_ value of 2. Five-fold serial dilutions of each strain were made with sterile water in 96-well plates and 2 μl of each dilution was spotted onto YPD plates with or without 0.035% MMS (Sigma-Aldrich, Cat# 129925). Plates were incubated at 30 °C for 2 days before being photographed.

### Statistical analysis

At least three samples (biological replicates or technical replicates) prepared from each strain per condition were used for statistical analysis to ensure adequate power for all studies. Statistical analysis was carried out by one-way or two-way *ANOVA* with multiple testing correlation within multiple groups or two-tailed Student’s *t* test within two groups. Data were presented as mean ± SD *p*< 0.05 is considered significant.

## Data availability

All data are contained within this manuscript.

## Supporting information

This article contains supporting information.

## Conflict of interest

The authors declare that they do not have any conflicts of interest with the content of this article.

## Figures and Tables

**Figure S2:**