# Supplementary material for: The Pof1 nicotinamide mononucleotide adenylyl transferase has a non-canonical role in NAD+ metabolism in the budding yeast Saccharomyces cerevisiae
Source: J Biol Chem. 2026 Apr 16;302(6):111456. doi: 10.1016/j.jbc.2026.111456 (PMC13191266; doi:10.1016/j.jbc.2026.111456)
Supplement: Supplementary Figure Legends [file mmc1.pdf]

**Supplementary Figure 1: Characterization of the expression of NMNATs in different nutrient conditions.**

*A*, Western blot analysis of each NMNAT in specific nutrient-deprived conditions. HA-tagged cells were cultured in standard SC or SC lacking specific nutrients for 6 hours. **Abbreviations**: -NA, low-nicotinic acid; -N, low-nitrogen source (ammonium sulfate); -Pi, low-phosphate; glu, glucose; SC, synthetic complete medium. *B*, quantitative analysis of the results of three sets of independent experiments including the one shown in *A*. Relative protein expression is normalized to Pgk1, and the protein expression in SC (Ctrl) is set to 1. Results show that compared with Nma1 and Nma2, Pof1 protein is more sensitive to glucose deprivation. *C*, gene expression analysis of each *NMNAT* in specific nutrient-deprived conditions determined by qPCR. Relative gene expression is normalized to *TAF10* and the gene expression in SC (Ctrl) is set to 1. For *B*, error bars represent data from three biological replicates. For *C*, the graphs are representative of the trend observed across four independent experiments representing four biological replicates. Error bars represent data from three technical replicates. The *p* values are calculated using One-Way *ANOVA.* (*, $p<0.05$; **, $p<0.01$; ***, $p<0.005$; ns, not significant)

**Supplementary Figure 2: NR supplementation alleviates MMS sensitivity caused by NR and $NAD^+$ deficiency.**

*A*, both *pho4*Δ and *npt1*Δ mutants display a decrease in the released and intracellular NR levels. *B*, a significant decrease in the $NAD^+$ (H) levels is observed in the *npt1*Δ mutant but not in the *pho4*Δ mutant. *C*, *npt1*Δ and *pho4*Δ cells are more sensitive to MMS. NR significantly enhances MMS resistance in WT, *npt1*Δ and *pho4*Δ cells. Serial dilutions (five-fold) of the indicated strains were spotted on YPD plate with or without 0.035% MMS and 10 µM NR. Cells were grown on YPD plates at 30 °C for 2 days. The experiments were repeated three times with a total of six biological replicates. Images shown are representative of the trend observed. For *A* and *B*, graphs are representative of the trend observed across three independent experiments representing three (for *A*) or six (for *B*) biological replicates. For *A*, error bars represent data from three technical replicates. For *B*, error bars represent data from two biological replicates, each with two technical replicates. The *p* values are calculated using One-Way *ANOVA* for *A,* and Two-Way *ANOVA* for *B*. (*, $p<0.05$; **, $p<0.01$; ***, $p<0.005$; ns, not significant)
